# A Multi-Network Approach Identifies Proteins Related to Dendritic Spines in Alzheimer’s Disease

**DOI:** 10.1523/ENEURO.0468-25.2026

**Published:** 2026-04-10

**Authors:** Emma L. Hobby, Audrey J. Weber, Evan Liu, Cheyenne Hurst, Kelsey M. Greathouse, Chris Gaiteri, Nicholas T. Seyfried, Jeremy H. Herskowitz

**Affiliations:** ^1^Department of Neurology, Killion Center for Neurodegeneration and Experimental Therapeutics, University of Alabama at Birmingham, Birmingham, Alabama 35294; ^2^Department of Biochemistry, Emory University School of Medicine, Atlanta, Georgia 30322; ^3^Department of Psychiatry, SUNY Upstate Medical University, Syracuse, New York 13210

**Keywords:** Alzheimer's disease, dendritic spines, human neuroscience, network analysis, proteomics, synapse

## Abstract

Proteomic studies have generated robust assessments of protein abundance changes in Alzheimer's disease (AD); however, identifying how the protein abundance changes affect specific biological processes remains a challenge. To address these hurdles, we used a multi-network computational analysis approach that integrated dendritic spine morphometry data with mass spectrometry-based proteomics from the same individuals. The samples exhibited a range of AD neuropathology and were categorized into three groups: controls, asymptomatic AD, and AD cases. Multiplex tandem mass tag mass spectrometry proteomic data (*N* = 8,212 proteins) was generated on Brodmann area 46 (BA46) dorsolateral prefrontal cortex (DLPFC) human samples (*N* = 41, 23 males and 18 females), from which dendritic spine morphometry analysis existed. To integrate the multi-scale data types, two computational network analysis methods were performed, including weighted coexpression network analysis (WGCNA) and SpeakEasy2 (SE2). Both WGCNA and SE2 revealed that the mitochondria protein modules were decreased in AsymAD and AD cases compared with controls, whereas the DNA repair modules were increased in AsymAD and AD compared with controls. Synaptic protein modules that correlated to multiple spine morphology traits were identified in both WGCNA and SE2. Pearson’s correlation analyses identified over a dozen individual proteins linked to multiple dendritic spine density and morphology traits. Collectively, these findings demonstrate how integration of spine morphometry data with proteomics can contextualize proteins for functional validation and identify synaptic alterations in AD progression.

## Significance Statement

Cognitive decline in Alzheimer's disease associates strongly with synapse and dendritic spine loss than amyloid-beta or tau pathology. However, one in three individuals harbor Alzheimer's disease neuropathology at death but were cognitively indistinguishable from baseline in life. Preservation of spines and synapses is hypothesized to prevent cognitive decline in these individuals. Identifying the molecular drivers of synaptic changes in Alzheimer's disease could yield deeper understanding of disease progression. Here, we utilized two computational network approaches that integrated multi-scale data, including proteomics and dendritic spine morphometry from the same humans, to identify proteins relevant to synapses in Alzheimer's disease. Hundreds of proteins related to mitochondria, DNA repair, and signaling were associated with alterations in synapse structure and function in Alzheimer's disease.

## Introduction

Alzheimer's disease (AD) remains a considerable public health burden as the aging population continues to expand without effective disease-modifying therapies (Scheltens et al., 2021). This irreversible neurodegenerative disorder causes progressive neuronal loss, leading to a decline in cognitive functions. AD is defined by its hallmark pathologies: extracellular amyloid-beta (Aβ) plaques and intracellular tau neurofibrillary tangles (NFTs; Duyckaerts et al., 2009). However, cognitive impairment in AD correlates more strongly with synapse and dendritic spine loss than with Aβ or tau pathology (DeKosky and Scheff, 1990; Terry et al., 1991; Boros et al., 2017; Chen et al., 2018). While cognitive impairment in AD is the loss of neuronal connectivity in brain regions crucial for memory functions, approximately one in three individuals harbor extensive AD neuropathology at death, but exhibit little to no cognitive impairment in life (Bennett et al., 2006; Driscoll and Troncoso, 2011; Sperling et al., 2011). These cognitively normal, or asymptomatic AD (AsymAD), individuals may offer an understanding of resilience to AD neuropathology or provide insight into the early mechanisms of disease pathogenesis.

Dendritic spine morphology is inseparably linked to synaptic function, as spines serve as the major postsynaptic sites for excitatory synapses in the brain. These actin-rich structures are highly plastic and alter their size and shape in relation to synaptic activity (Matsuzaki et al., 2001; Kasai et al., 2003; Hayashi and Majewska, 2005). Spines are classified based on their three-dimensional morphology as stubby, mushroom, thin, or filopodia (Chang and Greenough, 1984; Harris et al., 1992; Hering and Sheng, 2001). Previous reports showed that dendritic spine density in Brodmann area 46 (BA46) dorsolateral prefrontal cortex (DLPFC) is reduced in human aging and AD (Boros et al., 2019, 2017). Moreover, spine morphology is altered across AD progression in the DLPFC and temporal cortex. Spine length is increased significantly in AsymAD cases compared with AD and controls in the DLPFC (Boros et al., 2017), and increased spine head diameter in the inferior temporal gyrus is associated positively with episodic memory in aging and AD (Walker et al., 2024). Furthermore, thin and mushroom spine populations are lost in AD cases but maintained in controls and AsymAD, implicating their role in maintaining cognitive function despite burdening AD neuropathology (Boros et al., 2017). Identifying the molecular drivers of spine morphological changes in AD and AsymAD cases could yield a deeper understanding of how and why spine structure is altered in disease progression.

Proteomic studies can generate robust assessments of protein abundance alterations across disease conditions, such as AD (Seyfried et al., 2017; Yu et al., 2018). However, contextualizing the protein changes with biologically relevant cellular processes, such as dendritic spine morphology, remains a challenge. To address this, we used a multi-network computational approach that integrates and analyzes dendritic spine morphology traits with mass spectrometry (MS)-based proteomics from the same human brain samples. Network-based approaches extend beyond differential expression analysis by constructing coexpression networks that provide a system-wide view on protein abundance, revealing functionally related protein clusters associated with distinct biological processes (Ping et al., 2018). In the current study, we employed weighted coexpression network analysis (WGCNA) and SpeakEasy2 (SE2). The WGCNA clustering method groups highly correlated proteins into modules based on their abundance pattern across all samples (Zhang and Horvath, 2005; Langfelder and Horvath, 2008). Conversely, SE2 groups proteins into coabundance modules using an iterative consensus approach that identifies stable associations across multiple clustering rounds (Gaiteri et al., 2023). In the current study, we conducted MS-based proteomics on the same controls, AsymAD, and AD BA46 DLPFC human brain samples that were used for dendritic spine morphometry analyses in previous reports (Boros et al., 2017, 2019). By integrating dendritic spine morphometry data into two different computational networks, we identified proteins that may be linked to synaptic structure and function in AD.

## Materials and Methods

### Brain tissue samples and case classification

Postmortem human brain tissue samples from BA46 DLPFC were obtained from Emory University Alzheimer's Disease Research Center (*N* = 41, 23 human males and 18 human females; Extended Data Fig. 1-1; Boros et al., 2017, 2019). Cases exhibited a range of AD pathology and were categorized into three diagnostic groups: controls, AsymAD, and AD cases. Controls were defined as cognitively normal without AD pathology, AsymAD cases were cognitively normal with low to moderate AD pathology, and AD cases displayed severe cognitive impairment and high AD pathology. The case classification was based on three cognitive and neuropathology scores, including Mini-Mental State Examination (MMSE), Consortium to Establish a Registry for Alzheimer's Disease (CERAD), and Braak staging of neurofibrillary tau pathology.

The MMSE score is a widely used screening method for dementia severity that provides a global measure of cognitive function (Balsis et al., 2015). Scoring ranges from 0 to 30, with 30 indicating no cognitive impairment. AsymAD cases were no lower than 27, and severe to moderate AD cases had a range of scores from 10 to 20. It should be noted that end stages of the disease often preclude testing due to severity. Neuritic and diffuse plaques were scored semiquantitatively according to CERAD methods (0, A–C, or none, sparse, moderate, frequent; Mirra et al., 1991). AsymAD cases had moderate to frequent plaques, and AD cases had frequent plaques based on CERAD scoring. Braak staging (0-VI) was used to score tau neurofibrillary tangle accumulation. AsymAD cases had neurofibrillary tangle accumulation ranging from Braak stage 0-IV. AD cases ranged from I to VI; however, the majority of cases were within the IV–VI range. Global AD pathology burden was measured (none, low, intermediate, high) using the Amyloid Braak CERAD (ABC) score (Montine et al., 2012). AsymAD cases had pathology scores of low to intermediate on the ABC score. AD cases had pathology scores ranging from low to high; however, the majority of cases were in the intermediate to high range (Extended Data Fig. 1-1). The majority of cases contained no coexisting pathologies, such as stroke or Lewy bodies.

### Brain tissue homogenization

Sample homogenization was performed as previously described (Johnson et al., 2022; Hurst et al., 2023). Approximately 100 mg (wet tissue weight) of brain tissue was homogenized in 8 M urea lysis buffer (8 M urea, 10 mM Tris, 100 mM NaH_2_PO_4_, pH 8.5) with HALT protease and phosphatase inhibitor cocktail (Thermo Fisher Scientific) using a Bullet Blender (Next Advance). Each RINO sample tube (Next Advance) was supplemented with ∼100 μl of stainless-steel beads (0.9–2.0 mm blend, Next Advance) and 500 μl of lysis buffer. Immediately after excision, the tissue was homogenized with the Bullet Blender at 4°C and blended twice for 5 min intervals. After transferring the lysates to new Eppendorf LoBind tubes, three cycles of sonication were performed, consisting of 5 s of active sonication at 30% amplitude, followed by 15 s on ice. Then the samples were centrifuged for 5 min at 15,000 × *g*, and the supernatant was transferred to a new tube. Protein concentration was determined by bicinchoninic acid assay (Pierce) and one-dimensional SDS-PAGE gels were run, followed by Coomassie blue staining, as quality control for protein integrity and equal loading prior to protein digestion.

### Protein digestion

Protein digestion was conducted as previously described (Hurst et al., 2023). Then, 100 μg of each brain homogenate was aliquoted, and volumes were normalized with urea lysis buffer. Samples were reduced with 1 mM dithiothreitol at room temperature for 30 min, followed by 5 mM iodoacetamide alkylation for 30 min in the dark. Lysyl endopeptidase (Wako) at 1:100 (wt/wt) was added, and digestion was allowed to continue overnight. Samples were diluted sevenfold with 50 mM ammonium bicarbonate, and trypsin (Promega) was added at 1:50 (wt/wt). Further digestion was carried out for 16 h. The peptide solutions were then acidified to a final concentration of 1% (vol/vol) formic acid (FA) and 0.1% (vol/vol) trifluoroacetic acid (TFA) and desalted with a 30 mg hydrophilic-lipophilic balance column (HLB, Oasis). Each HLB column was first rinsed with 1 ml of methanol, washed with 1 ml of 50% (vol/vol) acetonitrile (ACN), and equilibrated twice with 1 ml of 0.1% (vol/vol) TFA. The samples were loaded onto the HLB column and washed twice with 1 ml of 0.1% (vol/vol) TFA. Elution was conducted with 2 volumes of 0.5 ml of 50% (vol/vol) ACN. An equal amount of peptide was aliquoted from each sample and combined to generate the pooled global internal standard (GIS), which was divided and labeled in each tandem mass tag (TMT) batch as described below. The eluates were then dried to completeness using a SpeedVac (Labconco).

### Tandem mass tag peptide labeling

Cases were first randomized by covariates (age, sex, diagnosis) into three batches. Then, peptides from each individual case and the GIS pooled standard or bridging sample (at least one per batch) were labeled using the TMT 11-plex kit (Thermo Fisher Scientific 90406). Labeling was performed as previously described (Hurst et al., 2023). Up to two TMT channels in each batch were used to label the GIS standards, and the remaining TMT channels were reserved for individual samples after randomization. In short, each sample (100 μg of peptides each) was resuspended in 100 mM triethylammonium bicarbonate buffer (100 μl). The TMT labeling reagents (5 mg), equilibrated to room temperature, were added to each channel along with anhydrous ACN (256 μl). After gently vortexing each channel for 5 min, 41 μl from each TMT channel was transferred to the peptide suspensions and incubated for 1 h at room temperature. The reaction was quenched with 5% (vol/vol) hydroxylamine (8 μl; Pierce), and all channels were then combined and dried by SpeedVac (Labconco) to ∼150 μl. Then they were diluted with 1 ml of 0.1% (vol/vol) TFA and acidified to a final concentration of 1% (vol/vol) FA and 0.1% (vol/vol) TFA. Labeled peptides were desalted with a 200 mg C18 Sep-Pak column (Waters). Each Sep-Pak column was activated with 3 ml of methanol. Then each column was washed with 3 ml of 50% (vol/vol) ACN and equilibrated twice with 3 ml of 0.1% TFA. The samples were then loaded, and each column was washed twice with 3 ml of 0.1% (vol/vol) TFA, followed by 2 ml of 1% (vol/vol) FA. Elution was performed with 2 volumes of 1.5 ml of 50% (vol/vol) ACN. The eluates were then dried to completeness using a SpeedVac.

### High-pH offline fractionation

Fractionation was performed as previously described (Hurst et al., 2023). Dried samples were resuspended in high-pH loading buffer (0.07% v/v NH_4_OH, 0.045% v/v FA, 2% v/v ACN) and loaded onto an Agilent ZORBAX 300 Extend-C18 column (2.1 mm × 150 mm with 3.5 µm beads). To carry out the fractionation, an Agilent 1100 HPLC system was used. Solvent A comprised 0.0175% (vol/vol) NH_4_OH, 0.01125% (vol/vol) FA, and 2% (vol/vol) ACN; solvent B comprised 0.0175% (vol/vol) NH_4_OH, 0.01125% (vol/vol) FA, and 90% (vol/vol) ACN. The sample elution was conducted over a 58.6 min gradient with a flow rate of 0.4 ml/min. The gradient comprised 100% solvent A for 2 min, then 0–12% solvent B over 6 min, then 12–40% over 28 min, then 40–44% over 4 min, then 44–60% over 5 min, and then held constant at 60% solvent B for 13.6 min. A total of 96 individual equal volume fractions were collected across the gradient and subsequently pooled by concatenation (Mertins et al., 2018) into 24 fractions and dried to completeness using a SpeedVac (Labconco).

### Liquid chromatography-tandem mass spectrometry

Equal volume of loading buffer (0.1% FA, 0.03% TFA, 1% ACN) was used to resuspend all fractions and analyzed by liquid chromatography-tandem mass spectrometry (LC-MS/MS) as previously described (Wingo et al., 2017; Johnson et al., 2022; Hurst et al., 2023). Peptide eluents were separated on a self-packed C18 (1.9 μm, Dr. Maisch) fused silica column (25 cm  ×  75 μM internal diameter, New Objective) by a Dionex UltiMate 3000 RSLCnano liquid chromatography system (Thermo Fisher Scientific). An Orbitrap Fusion mass spectrometer was used to monitor the peptides (Thermo Fisher Scientific). Sample elution was performed over a 120 min gradient with flow rate of 300 nl min^−1^ with buffer B ranging from 1 to 50% (buffer A, 0.1% FA in water; buffer B, 0.1% FA in 80% ACN). The mass spectrometer was set to acquire in data-dependent mode with a cycle time of 3 s, using the top speed workflow. Each cycle consisted of one full scan followed by as many MS/MS (MS2) scans that could fit within the time window. Full MS scans were collected at a resolution of 120,000 [400–1,400 m/z range, 4 × 10^5^ Automatic Gain Control (AGC), 50 ms maximum ion injection time]. All higher-energy collisional dissociation MS/MS spectra were acquired at a resolution of 60,000 (1.6 m/z isolation width, 35% collision energy, 5 × 10^4^ AGC target, 50 ms maximum ion time). Dynamic exclusion was set to exclude previously sequenced peaks for 20 s within a 10 ppm isolation window.

### Database searching and protein quantification

Database searching and protein quantification was performed as previously described (Hurst et al., 2023). All raw MS data files (generated across three batches) were analyzed in the Proteome Discoverer software suite (version 2.3, Thermo Fisher Scientific) and MS/MS spectra were searched against the UniProtKB human proteome database. The Sequest HT search engine was used with the following parameters: fully tryptic specificity; maximum of two missed cleavages; minimum peptide length of 6; fixed modifications for TMT tags on lysine residues and peptide N-termini (+229.162932 Da) and carbamidomethylation of cysteine residues (+57.02146 Da); variable modification for oxidation of methionine residues (+15.99492 Da) and deamidation of asparagine and glutamine (+0.984 Da); precursor mass tolerance of 20 ppm; and fragment mass tolerance of 0.05 Da. The Percolator node was used to filter to a false discovery rate (FDR) of <1% for the peptide spectral matches (PSMs). Peptides were assembled into proteins following spectral alignment and further filtered based on the combined probabilities of their constituent peptides to a final FDR of 1%. To achieve parsimony across individual batches, multi-consensus was performed. In cases of redundancy, shared peptides were assigned to the protein sequence in adherence with the principles of parsimony. Reporter ions were quantified from MS2 scans using integration tolerance of 20 ppm with the most confident centroid setting. Quantification was restricted to PSMs with <50% isolation interference, and only unique or razor (i.e., parsimonious) peptides were included.

### Batch correction and data preprocessing

Conducted as previously described (Johnson et al., 2022; Hurst et al., 2023), only proteins quantified in >50% of samples across all three TMT batches were included in following analyses (*N* = 8,212 proteins). Log2 abundances were normalized as a ratio dividing by the central tendency of pooled standards (GIS). Tunable Approach for Median Polish of Ratio (TAMPOR) was used for batch correction (https://github.com/edammer/TAMPOR), which is an iterative median polish algorithm for removing technical variance across batches (Johnson et al., 2022). To visualize batch contributions to variation before and after correction, multidimensional scaling (MDS) plots were used. Network connectivity was used to remove outliers, that is, samples that were >3 standard deviations away from the mean (Johnson et al., 2022). Lastly, nonparametric bootstrap regression was performed to remove the potentially confounding covariates of batch and postmortem interval (PMI). Each trait was subtracted times the median coefficient from 1,000 iterations of fitting for each protein, while protecting for case diagnosis (Control, AsymAD, AD).

### Weighted coexpression network analysis

We used the WGCNA (version 1.73) algorithm to generate a central network of coexpression modules as previously described (Langfelder and Horvath, 2008; Hurst et al., 2023). The WGCNA::blockwiseModules function was run with soft-threshold power at 8.0, deepSplit of 4, minimum module size of 30, merge cut height at 0.07, mean topological overlap matrix (TOM) denominator, using biweight midcorrelations (bicor), signed network type, pamStage and pamRespectsDendro parameters both set to TRUE, and a reassignment threshold of 0.05. This function calculates the pairwise bicor between protein pairs. The resulting correlation matrix was converted into a signed adjacency matrix and subsequently used to calculate a TOM, which represents pairwise protein expression similarity across samples. Hierarchical clustering based on 1 minus TOM, followed by dynamic tree cutting, was used to identify protein modules. For each module, an eigenprotein (ME) value was defined and represented the module's collective abundance pattern and covariance structure. Pearson’s correlation between proteins and MEs was used as a module membership measure, defined as kME.

### SpeakEasy2 (SE2) network analysis

We used the R-version of the SE2 (version 0.1.8) algorithm to construct a protein coexpression network as previously described (Gaiteri et al., 2023; Ng et al., 2024). Protein abundance levels across each sample were used to estimate the degree of interaction between each pair of proteins. This estimation was repeated to generate a protein correlation matrix, which was then analyzed using SE2 to identify nine protein modules. Proteins are assigned through a dynamic process in which initial random communities gain node members to which they are most specifically connected. After a pool of partitions has been generated in this way, from different (random) initial conditions, the final partition that is selected as the final solution is that with the highest average NMI to all other partitions. The purpose of this is to avoid selecting an outlying solution and to pick one which is broadly representative. To increase robustness, SpeakEasy2 essentially has no manual parameter settings but allows the dynamics node assignment to select the number of clusters. Since some datasets may have hierarchical clusters, there is the ability to reprocess data - to subcluster or sub-subcluster data.

### Gene ontology (GO)

To characterize differentially expressed proteins and coexpressed proteins based on GO annotation, we used GO Elite (version 1.2.5) as previously described (McKenzie et al., 2017; Seyfried et al., 2017; Johnson et al., 2022; Hurst et al., 2023; Walker et al., 2023). Ontology terms were pruned using the *z*-score, and a Fisher exact test was used for overrepresentation analysis (ORA). The *z*-score cutoff for initial filtering was set at 1.96, and the permuted *p* value cutoff was set to 0.05. The minimum number of changed proteins was limited to 5 and the number of permutations for ORA = 2,000.

### Golgi-Cox staining

BA46 DLPFC human tissue samples were obtained from the Emory Alzheimer's Disease Research Center (Extended Data Fig. 1-1). The same cases were used for both dendritic spine morphometry analysis and proteomics. Tissue samples were processed and stained as previously described (Boros et al., 2017; Boros et al., 2019; Walker et al., 2023). Immediately following dissection, all tissue samples were fixed in 4% paraformaldehyde and stored in preservative solution containing sodium azide at 4°C. Tissue blocks of ∼20 × 20 × 5 mm were sectioned into 250 µm slices (∼15 per block) using a Leica vibratome (VT1000S, Leica Biosystems) and stored in preservative solution (0.1% wt/vol sodium azide in phosphate-buffered saline) until Golgi-Cox impregnation. All tissues were stained using the FD Rapid Golgi Stain Kit (catalog #PK401, FD NeuroTechnologies), following the manufacturer's instructions with the following modifications. Tissue slices were saturated in a chromate mixture of Solution A (potassium dichromate and mercuric chloride) and Solution B (potassium chromate). The chromate solution was replaced after the first 24 h, and the tissue was then left in chromate solution in the dark for 6 weeks. Next, tissue slices were immersed in Solution C for 48 h, and this solution was replaced after 24 h. Next, tissues were plated on 75 × 25 mm gelatin-coated slides (catalog #PO101, FD NeuroTechnologies) using additional Solution C and allowed to dry for 2 h in the dark. Tissues were then submerged sequentially in mixtures of Solution D (from kit), Solution E (from kit), and distilled water according to the manufacturer's instructions. Tissues were dehydrated with graded alcohols (70, 90, and100% ethanol in deionized water) after rinsing with distilled water and cleared with xylenes (catalog #X3P-1GAL, Thermo Fisher Scientific). Slides were sealed with Permount Toluene Solution (catalog #SP15-100, Fisher Chemicals) and coverslipped with spacers (SecureSeal Spacer, 20 mm diameter × 0.12 mm depth, catalog #70327-205, Electron Microscopy Sciences) and 50 × 24 mm glass (cover glass, rectangles, 24 × 50 mm, thickness = 0.13-0.17 mm, catalog #633153, Carolina Biological). Opaque slide holders (catalog #12-587-10, Fisher Scientific) were used to store the slides at room temperature.

### Bright-field microscopy

Microscopy was performed by blinded experimenters as previously described (Walker et al., 2023). Dendrites on layers II and III pyramidal neurons in BA46 gray matter were imaged. For each case, many tissue slices were Golgi stained, and from each tissue slice, two or more neurons were imaged and analyzed. Ten to twenty Golgi-stained cells were sampled per case. Each slide contained multiple slices of Golgi-stained tissues from the same original block of tissue, and two or more neurons minimum were imaged from each slice of tissue. The amount of slices imaged per slide was not constrained by PMI; however, the AD patient group was occasionally constrained due to the expected neuronal loss in AD. More tissue slices were required to be powered in the AD cases as they had fewer total Golgi-stained neurons. From each neuron, one dendritic segment was selected for imaging. Neurons were considered eligible for analysis if they (1) were positioned near the center of the tissue depth, (2) were free of major staining artifacts or debris, and (3) were fully impregnated. For neurons meeting these criteria, a single dendritic branch was imaged. Dendrites were required to (1) be unobstructed, isolated, and nonoverlapping with neighboring dendrites, (2) exceed 30 µm in length, and (3) be ∼1 µm in diameter. To establish the region of interest, each tissue slice was initially viewed under 10× magnification. Then a dendrite within the region of interest was viewed at 60× magnification and evaluated against the selection criteria above. *Z*-stacks were captured with a *z*-step size of 0.1 µm at 60× using a Nikon Plan Apo 60×/1.40 NA oil-immersion objective on a Nikon Eclipse Ni upright microscope with a Lumen 200 light source and Nikon DS-43 Digital Sight for bright-field microscopy. Images were captured using the following parameters: lamp, 100%; field stop, 1.5 mm; exposure, 60 ms; analog gain, 2.0–2.4×; image size, 1,028 × 1,028 px.

### Dendritic spine morphometry analysis

Dendrite and dendritic spine reconstructions were conducted by blinded experimenters as previously described (Boros et al., 2017, 2019; Walker et al., 2023). Image stacks of neuronal dendrites were converted to 16 bit TIFF files in ImageJ and then imported to Neurolucida 360 (2.70.1, MBF Bioscience). Images were first assessed for quality. Those with dendritic spines that were difficult to discern were excluded from analysis. The blinded experimenter would decide, after the image was in Neurolucida software, whether the dendrite and spines were suitable for analysis. Diagnosis or PMI did not influence occasional poor image quality. Dendrites were traced using a semiautomated directional kernel algorithm. Spines were traced using voxel clustering. Initiation and termination points for dendrite reconstruction were established using the following criteria: (1) ≥10 µm away from the distal tip of the dendrite, (2) consistent diameter, (3) level axis with limited movement in the *z* plane, and (4) ≥30 µm in length. Next, the experimenters manually examined each assigned point in the *x*, *y*, and *z* planes to verify that the points were located on the dendrite and were not artificially assigned. The dendrite was first viewed at *x*–*z* or *y*–*z* planes to ensure that points were correctly positioned at the midline of the dendrite. Points were then verified in the *x*–*y* plane, and the diameter of the reconstruction at each point was confirmed to match the dendrite diameter. Dendritic spine reconstruction utilized the following parameters for classification: outer range, 7 µm; minimum height, 0.3 µm; detector sensitivity, 90–125%; minimum count, 8 voxels. The morphology of each reconstructed spine was examined to verify that axial smear did not cause misrepresentation, and the merge and slice tools were used to correct inaccuracies. The position of each spine backbone point was confirmed by the experimenter. To correct a misrepresentative backbone, the spine was viewed from the *z* plane, and experimenters moved backbone points in the *x*–*y* plane. Any repositioning in the *x*–*z* or *y*–*z* plane was performed while the spine was being viewed from the lateral angle. Morphometric analysis was conducted as previously described (Boros et al., 2017; Boros et al., 2019; Walker et al., 2023) for each spine. Spines were categorized into thin, stubby, mushroom, and filopodia classes. The following established parameters were used for spine classification: head-to-neck ratio, 1.1; length-to-head ratio, 2.5; mushroom head size, 0.35 µm; filopodium length, 3.0 µm. Spines with a head-to-neck ratio >1.1 and head diameter >0.35 µm were classified as mushroom. Spines were classified as filopodia or thin if head-to-neck ratio was <1.1 and either (1) length-to-head ratio was >2.5 or (2) head size was <0.35 µm. Of these, if the total length was >3.0 µm, the spine was classified as filopodia, and if <3.0 µm, as thin. Reconstructions were exported to Neurolucida Explorer (2.70.1, MBF Bioscience), where data were collected for quantitative analysis. The parameters for dendritic spine measurement included length, head diameter, and volume. These parameters were exported and collected in Microsoft Excel. Derived measurements, such as spine density, were calculated from raw measurement data. Spine density was calculated by determining the number of spines per 10 µm of dendrite length. Spine length was defined as the curvilinear backbone length from the insertion point to the most distal point of the spine head. Head diameter was defined as the breadth of the spine head at its widest cross-sectional point. Spine volume was measured by the number of voxels that make up the spine object multiplied by the volume of a single voxel. The volume of a single voxel is *x* resolution × *y* resolution × *z* resolution. In total 434 neurons and 4,558 individual dendritic spines were analyzed. Approximately 119 spines per control case, 109 spines per AsymAD case, and 95 spines per AD case were analyzed (Extended Data Fig. 1-2).

### Statistical analysis

All proteomic statistical analyses were performed in R (version 4.4.2). The box plots represent the median and 25th and 75th percentile extremes, and the hinges of a box represent the interquartile range of the two middle quartiles of data within a group. Error bars extents are defined by the farthest data points up to 1.5 times the interquartile range away from the box hinges. Correlations were performed using the biweight midcorrelation function from the WGCNA package. Group comparisons in human brain samples were performed with one-way ANOVA with Tukey's post hoc correction of all comparisons.

Pairwise Pearson’s correlations were computed between normalized protein abundance values and cognitive scores, AD neuropathology, and dendritic spine density and morphology traits using the rcorr() function from the Hmisc R package. Resulting correlation coefficients (*r*) and *p* values were extracted and adjusted for multiple comparisons using both the Benjamini–Hochberg (BH) procedure (FDR threshold = 0.10) and Storey's *q* value estimation (R package *q* value). Proteins with *q* < 0.05 were considered statistically significant. For visualization, the top 10 proteins per trait were ranked by *q* value, regardless of correlation direction. Full correlation statistics are provided in Extended Data Fig. 6-1. All analyses were performed using Windows 11 (version 25H2).

### Code accessibility

The code described in the paper is freely available online at https://github.com/ehobby33/eNeuro-repo.git. The code is available as Extended Data 1.

10.1523/ENEURO.0468-25.2026.d1 Data 1 WGCNA and SE2 code. The WGCNA and SE2 network analysis code described in the paper can be accessed in Extended Data.zip file. The code constructs proteomic coexpression network analysis, module detection, hub protein identification, and module-trait correlations. Download Data 1, ZIP file.

## Results

### Aβ42 peptide measurements align with AD neuropathology and cognitive scores

Brain tissue samples from BA46 DLPFC were obtained from Emory University Alzheimer's Disease Research Center (*N* = 41) and analyzed using multiplex TMT-MS (Extended Data Fig. 1-1). Proteomic samples were enzymatically digested with trypsin into peptides and labeled individually with isobaric TMT, followed by LC-MS/MS. Protein abundances were log2 transformed and normalized to the pooled GIS standards. TMT-MS quantified protein levels were filtered for missing values in <50% of samples and adjusted for technical variance, outlier removal, and confounding effects of covariates, including batch effects and PMI, for a final abundance dataset of 8,212 proteins. Cases were categorized into three groups: control, asymptomatic AD (AsymAD), and AD. These categories are based on semiquantitative measures of amyloid (CERAD), neurofibrillary tau deposition (Braak stage), and cognitive function near the time of death (MMSE). AsymAD cases exhibit accumulation of Aβ plaques and tau tangles similar to AD cases but without significant cognitive impairment, which is considered to be an early preclinical stage of AD (Jack et al., 2018).

**Figure 1.**
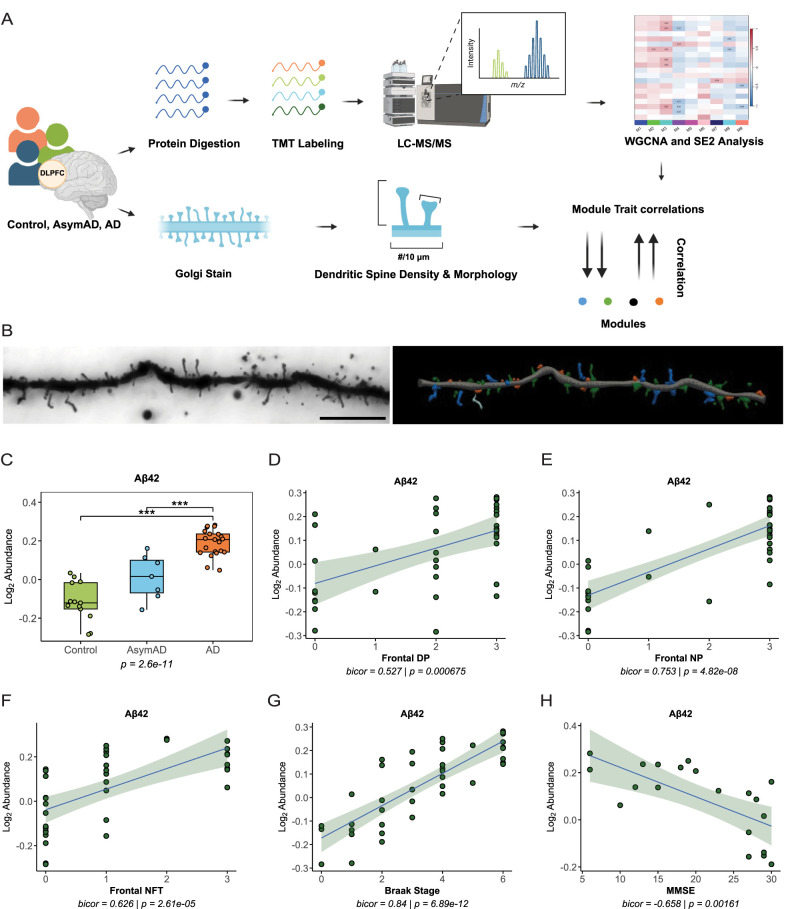
Aβ42 peptide measurements align with AD neuropathology and cognitive scores. ***A***, Schematic representation of experimental workflow for BA46 human brain tissue samples with matched dendritic spine morphometry analyses. ***B***, Representative 60× bright-field image of a Golgi-stained dendrite from BA46 (left) with digital 3D reconstruction (right). Scale bars, 10 μm. Green, thin spines; orange, stubby spines; dark blue, mushroom spines; light blue, filopodia. ***C***, Aβ42 normalized abundance was measured across controls, AsymAD, and AD cases. AD cases had significantly greater amounts of Aβ42 compared with controls and AsymAD cases. One-way ANOVA (*F*_(2,38)_ = 49.5, *p* = 2.6 × 10^−11^) with Tukey's multiple comparisons: controls versus AD, ***p = 1.71 × 10^−11^; AsymAD versus AD, ***p = 0.000126. ***D***, Aβ42 normalized abundance correlated positively with frontal diffuse plaques (DP). Line of best fit was determined using a linear model. The confidence interval is shaded around the line. ***E***, Aβ42 normalized abundance correlated positively with frontal neuritic plaques (NP). ***F***, Aβ42 normalized abundance correlated positively with frontal neurofibrillary tangles (NFT). DP, NP, and NFTs are ordinal variables that represent neuropathology burden as follows: 0 = none, 1 = sparse, 2 = moderate, and 3 = frequent. ***G***, Aβ42 normalized abundance correlated positively with Braak stage. ***H***, Aβ42 normalized abundance correlated negatively with Mini-Mental State Examination (MMSE). See Extended Data Figures 1-1, 1-2, and 1-3 for more details.

In parallel, dendritic spine density and morphology was measured in BA46 layers II and III pyramidal neurons from the same 41 individuals (Extended Data Fig. 1-2, Extended Data Fig. 1-3). WGCNA and SE2 were used to construct networks of coexpressed protein modules. The protein modules eigenprotein values were then correlated with AD neuropathology, cognitive scores, and dendritic spine traits (Fig. 1*A*,*B*). A key pathological hallmark of AD is abnormal accumulation of amyloid-β (Aβ) into extracellular plaques (Iwatsubo et al., 1994). To provide a qualitative test on our TMT proteomics, Aβ42 peptide abundance was compared across disease states. The normalized abundance of Aβ42 peptides tracked with disease state, where AD cases had significantly higher Aβ42 than controls and AsymAD cases had significantly lower Aβ42 than AD (Fig. 1*C*). Aβ42 peptide abundance was correlated positively with diffuse plaques, neuritic plaques, neurofibrillary tangles, and Braak score across all cases (Fig. 1*D–G*). Conversely, Aβ42 was correlated negatively with MMSE score (Fig. 1*H*). Collectively, these results indicated that our TMT-MS data reflects known disease hallmarks of AD.

10.1523/ENEURO.0468-25.2026.f1-1 Figure 1-1 **Clinical and Demographic Case Data.** Clinical and demographic case data from 41 postmortem human brain tissue samples from dorsolateral prefrontal cortex. Download Figure 1-1, XLS file.

**Figure 2.**
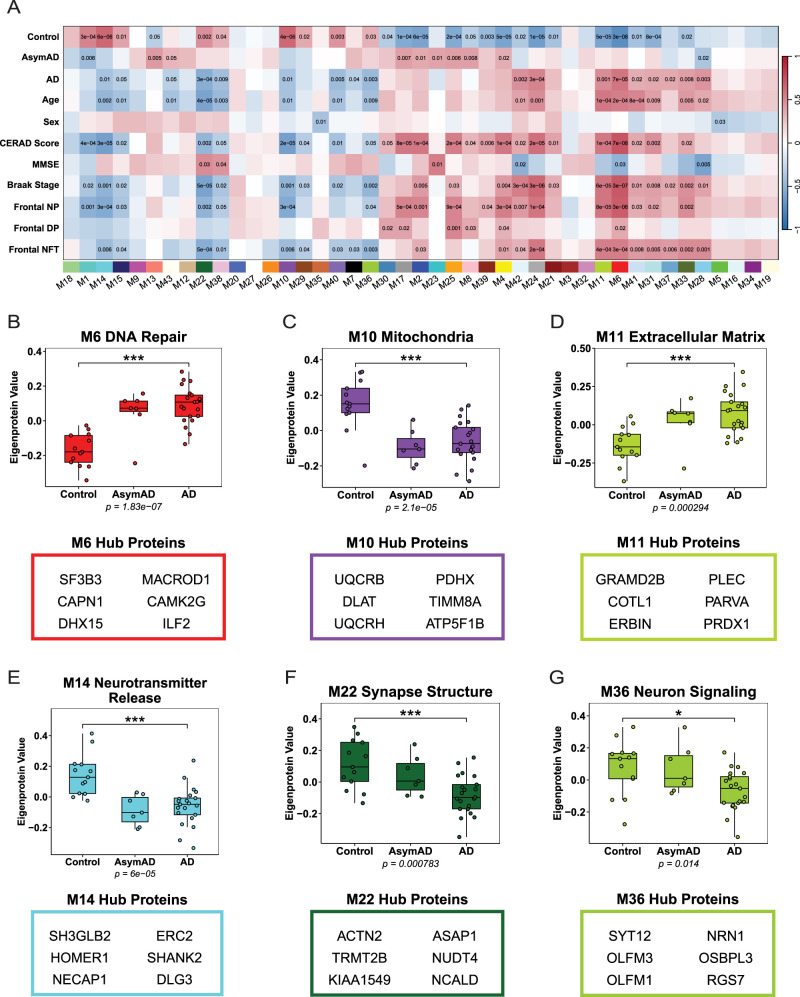
Weighted coexpression network analysis identifies modules associated with AD neuropathology. WGCNA network analysis was constructed with 8,212 proteins from *N* = 41 samples of BA46 DLPFC and yielded 43 modules. ***A***, Biweight midcorrelation (Bicor) for each module's eigenprotein abundance was correlated with case traits. Red indicates positive correlations. Blue indicates negative correlations. Stronger color intensity reflects strong correlation magnitude. Uncorrected *p* values are shown for correlations with *p* < 0.05. ***B***, Module 6 (M6) eigenprotein abundance was significantly higher in AD and AsymAD cases compared with control. One-way ANOVA (*F*_(2,38)_ = 23.99, *p* = 1.83 × 10^−7^) with Tukey's multiple comparisons: controls versus AD, ***p = 1.29 × 10^−7^. M6 GO analysis revealed terms associated with DNA damage response. ***C***, Module 10 (M10) was enriched with mitochondria-related GO terms. M10 eigenprotein expression was reduced in AD and AsymAD compared with controls. One-way ANOVA (*F*_(2,38)_ = 14.49, *p* = 2.10 × 10^−5^) with Tukey's multiple comparisons: controls versus AD, ***p = 5.20 × 10^−5^. ***D***, Module 11 (M11) eigenprotein abundance was elevated in AD and AsymAD compared with controls. One-way ANOVA (*F*_(2,38)_ = 10.15, *p* = 2.94 × 10^−4^) with Tukey's multiple comparisons: controls versus AD, ***p = 0.000193. M11 is described by GO terms related to the extracellular matrix. ***E***, M14 eigenprotein abundance was reduced in AD and AsymAD compared with controls. One-way ANOVA (*F*_(2,38)_ = 12.69, *p* = 5.99 × 10^−5^) with Tukey's multiple comparisons: controls versus AD, ***p = 0.000129. Module 14 (M14) was enriched with GO terms for neurotransmitter release. ***F***, Module 22 (M22) eigenprotein abundance was decreased in AD and AsymAD compared with controls. One-way ANOVA (*F*_(2,38)_ = 8.68, *p* = 0.000783) with Tukey's multiple comparisons: controls versus AD, ***p = 0.000651. GO terms for M22 were related to synapse structure. ***G***, Module 36 (M36) eigenprotein abundance was reduced in AD compared with controls and AsymAD. One-way ANOVA (*F*_(2,38)_ = 4.79, *p* = 0.01398) with Tukey's multiple comparisons: controls versus AD, *p = 0.0221. M36 GO terms were linked to neuron signaling. See Extended Data Figures 2-1, 2-2, and 2-3 for more details.

10.1523/ENEURO.0468-25.2026.f1-2 Figure 1-2 **Dendritic Spine Measurements Across Conditions.** Box plots of dendritic spine measurements across Control, AsymAD, and AD groups. One−way ANOVA with Tukey post hoc comparisons, and Grubbs’ test used for outlier detection. Download Figure 1-2, ZIP file.

**Figure 3.**
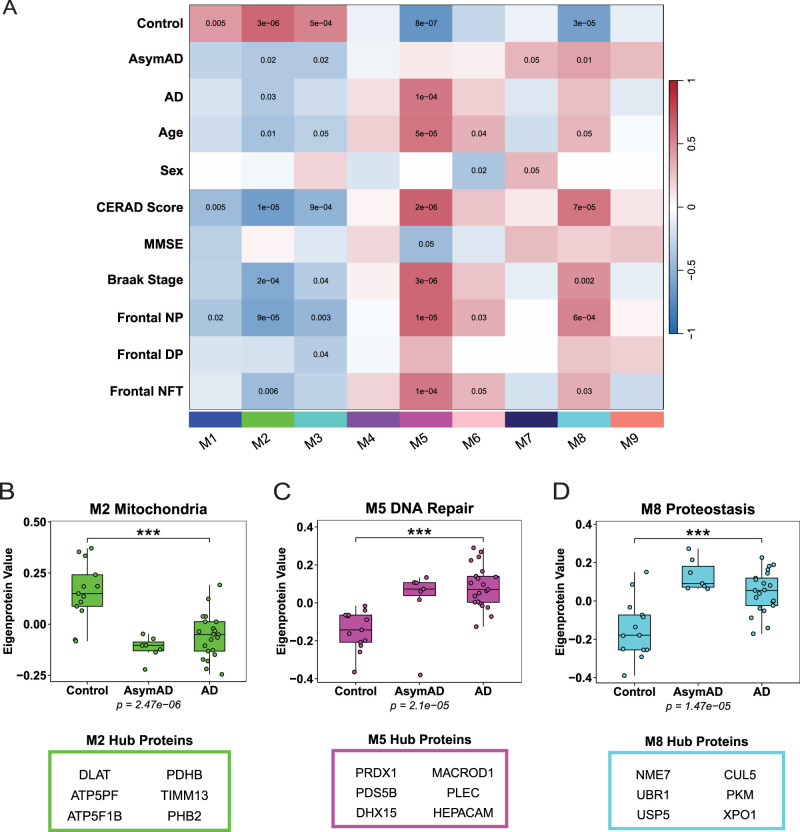
SpeakEasy2 network analysis identifies modules associated with AD neuropathology. SpeakEasy2 network analysis was constructed with 8,212 proteins from *N* = 41 BA46 DLPFC samples, yielding 9 protein modules. ***A***, Biweight midcorrelation (Bicor) for each module's eigenprotein abundance was correlated with case traits. Red indicates positive correlations. Blue indicates negative correlations. Stronger color intensity reflects strong correlation magnitude. Uncorrected *p* values are shown for associations with *p* < 0.05. ***B***, Module 2 (M2) is enriched with mitochondria-related GO terms. M2 eigenprotein abundance is reduced in AD compared with controls. One-way ANOVA (*F*_(2,38)_ = 18.49, *p* = 2.47 × 10^−6^) with Tukey's multiple comparisons: controls versus AD, ***p = 1.34 × 10^−5^. ***C***, Module 5 (M5) GO revealed terms associated with DNA repair mechanisms. M5 eigenprotein expression is increased in AD and AsymAD compared with controls. One-way ANOVA (*F*_(2,38)_ = 14.50, *p* = 2.10 × 10^−5^) with Tukey’s multiple comparisons: controls versus AD, ***p = 1.22 × 10^−5^. ***D***, Module 8 (M8) GO terms related to proteostasis. M8 eigenprotein expression is higher in AD and AsymAD compared with controls. One-way ANOVA (*F*_(2,38)_ = 15.13, *p* = 1.47 × 10^−5^) with Tukey's multiple comparisons: controls versus AD, ***p = 1.95 × 10^−4^. See Extended Data Figures 3-1, 3-2, 3-3, and 3-4 for more details.

10.1523/ENEURO.0468-25.2026.f1-3 Figure 1-3 **Dendritic Spine Data.** Dendritic spine density and morphology measurements from each human sample. Download Figure 1-3, XLS file.

### Weighted coexpression network analysis identifies modules associated with AD neuropathology

WGCNA groups proteins together based on their expression pattern across all samples (Langfelder and Horvath, 2008). This yielded 43 modules, each identified by color and number, ranked according to size from largest (M1, *N* = 1,389 proteins) to smallest (M43, *N* = 37 proteins). To assess how module biology relates to disease status and neuropathology scores, representative abundance profiles (eigenproteins) were calculated for each module. Correlations between case traits and modules were assessed using biweight midcorrelation (Fig. 2*A*), a median-based metric that is more robust to outliers (Song et al., 2012). The connectivity of proteins within a module was assessed by measuring the correlation of the expression pattern of one protein against the expression patterns of every other protein in the module (Extended Data Fig. 2-1). Hub proteins represent the strongest correlations to the collective expression pattern of that module (Extended Data Fig. 2-2). GO analysis identified biological processes, molecular functions, and cellular components enriched in each module. Representative module biology was determined using top GO terms for each module (Extended Data Fig. 2-3).

10.1523/ENEURO.0468-25.2026.f2-1 Figure 2-1 **WGCNA Module Assignments.** List of proteins assigned to each WGCNA module. Download Figure 2-1, XLS file.

10.1523/ENEURO.0468-25.2026.f2-2 Figure 2-2 **WGCNA Hub Protein Plots.** Box plots of WGCNA hub proteins, each colored with respective module color. Log2 abundance values across Control, AsymAD, and AD groups. Download Figure 2-2, ZIP file.

10.1523/ENEURO.0468-25.2026.f2-3 Figure 2-3 **WGCNA GO terms.** Gene ontology (GO) analysis was performed to gain insight into the biological meaning of each protein network module. Enrichment for a given ontology is shown by z score. Download Figure 2-3, ZIP file.

Six modules (M6, M10, M11, M14, M22, M36) correlated significantly with disease status and AD neuropathology. M6 eigenprotein abundance was significantly higher in AD and AsymAD cases compared with control. M6 GO analysis revealed that the module proteins were associated with DNA repair, containing hub proteins SF3B3, CAPN1, DHX15, MACROD1, CAMK2G, and ILF2 (Fig. 2*B*). DNA repair-associated proteins are increased in AD, aligning with prior evidence of impaired DNA repair mechanisms in AD (Lin et al., 2020; Walker et al., 2023). M10 eigenprotein abundance was reduced in AD and AsymAD cases compared with control. M10 contained hub proteins UQCRB, DLAT, UQCRH, PDHX, TIMM8A, and ATP5F1B that were related to mitochondria functions, including electron transport chain and cellular bioenergetic dynamics (Fig. 2*C*). Consistent with other findings, mitochondrial proteins are reduced in AD leading to increased oxidative stress and bioenergetic failure, collectively driving AD progression (Stauch et al., 2014; Johnson et al., 2022; Walker et al., 2023; D’Alessandro et al., 2025). M11 eigenprotein abundance was elevated in AD and AsymAD cases compared with control. M11 was enriched for extracellular matrix and cell adhesion processes, containing hub proteins GRAMD2B, COTL1, ERBIN, PLEC, PARVA, and PRDX1. GO analysis indicated that M11 proteins contribute to integrin-mediated interactions and cellular scaffolding components (Fig. 2*D*). Increased ECM proteins observed in AD and AsymAD aligns with prior evidence on the involvement of ECM dysfunction in AD (Seyfried et al., 2017; Walker et al., 2023). M14 eigenprotein abundance was reduced in AD and AsymAD cases compared with controls. M14 is enriched with GO terms for neurotransmitter release and contains hub proteins SH3GLB2, HOMER1, NECAP1, ERC2, SHANK2, and DLG3 (Fig. 2*E*). The reduction in synaptic protein abundance in this module likely reflects the loss of synaptic functions in AD (Forner et al., 2017; Seyfried et al., 2017; Walker et al., 2023). M22 eigenprotein abundance was increased in control cases compared with AD and AsymAD. M22 GO terms related to synapse structure, containing hub proteins ACTN2, TRMT2B, KIAA1549, ASAP1, NUDT4, and NCALD (Fig. 2*F*). M22 hub proteins play a role in cytoskeleton organization and calcium-dependent vesicle trafficking, which is consistent with impaired actin remodeling in AD (Penzes and Vanleeuwen, 2011; Seyfried et al., 2017). M36 eigenprotein abundance was significantly higher in control and AsymAD cases compared with AD. M36 contained hub proteins SYT12, OLFM3, OLFM1, NRN1, OSBPL3, and RGS7 and was characterized by GO terms related to neuron signaling (Fig. 2*G*). Increased abundance of M36 proteins in control and AsymAD aligns with previous findings on the role of these proteins in synaptic maintenance and resilience (Johnson et al., 2022; Hurst et al., 2023). Collectively, these modules highlight protein networks underlying mitochondrial, DNA repair, and synaptic processes relevant to AD.

### SpeakEasy2 network analysis identifies modules associated with AD neuropathology

To analyze the data using an orthogonal network approach, SE2 was utilized. Running multiple clustering methods allows experimenters to estimate the extent to which module correlations may be computational method specific. SE2 groups proteins into modules using an iterative consensus-based clustering method. Proteins that are consistently grouped together over multiple iterations are then clustered into protein coexpression modules (Gaiteri et al., 2023). Using the same 8,212 proteins from BA46 DLPFC, SE2 yielded 9 modules (M1-9), each identified by color and number, where M1 was largest (*N* = 1,254) and M4 was smallest (*N* = 565 proteins). Based on the difference in quantity of modules generated between WGCNA and SE2, we calculated the similarity in network clustering between WGCNA and SE2 by performing partition comparison analysis (Extended Data Fig. 3-1). WGCNA module structure is influenced by several parameters that determine network topology and module clustering. The soft-threshold power determines the weights of connecting protein pairs, which produces a weighted coexpression network where strong correlations are emphasized and weak correlations are punished (Zhang and Horvath, 2005; Horvath and Dong, 2008). As the soft-threshold power increases, greater weight is assigned to shorter geodesic distances relative to longer distances (Horvath and Dong, 2008). Another WGCNA clustering parameter is how the dendrogram branches are cut into clusters. This process uses dynamic tree cutting (or deepSplit) which controls how finely the protein modules are divided. Lower deepSplit settings produce larger modules, whereas higher settings result in more cluster division, yielding smaller modules (Langfelder et al., 2008; Langfelder and Horvath, 2008). Lastly, the merge cut height parameter determines the degree of similarity that is required between module eigenproteins for modules to be merged. Lower merge cut height thresholds preserve finer distinctions between modules, whereas higher thresholds result in merging of closely related modules (Langfelder and Horvath, 2008). Collectively these parameters shape the number and size of WGCNA modules.

To measure the cluster similarity between WGCNA and SE2, adjusted Rand index (ARI) and normalized mutual information (NMI) were calculated. ARI is a measure of the size of the cluster–cluster overlap between two partitions, while NMI is a measure of the amount of information one partition provides about another partition (Gaiteri et al., 2023). Partition similarity between WGCNA and SE2 was assessed across a range of soft-threshold powers, deepSplit values, and merge cut height thresholds (Extended Data Fig. 3-2). The mean ARI between the SE2 result was 0.353 ± 0.060 standard deviation (SD) and mean NMI was 0.466 ± 0.059 SD. Referencing the within-WGCNA results as a means of contextualizing these differences, we find the least similar WGCNA results had an ARI of 0.570 and NMI of 0.536 (Extended Data Fig. 3-2). ARI and NMI both decreased as WGCNA deepSplit values increased, indicating that WGCNA clustering that yields larger modules is more similar to SE2 clustering. Likewise, as WGCNA merge cut height thresholds increased, ARI and NMI increased, reflecting higher similarity to SE2 clustering. As soft-threshold power was increased across different WGCNA deepSplit values, there was a slight decrease in ARI and NMI.

Similar to WGCNA, representative abundance profiles or eigenproteins were calculated for each module from SE2. Using biweight midcorrelation, modules were correlated with case data and disease status (Fig. 3*A*). Module protein connectivity was assessed by measuring the correlation of the abundance pattern of one protein against the abundance patterns of all other proteins in the module (Extended Data Fig. 3-3). Hub proteins were considered most strongly correlated with the overall expression pattern of the module. GO analysis defined the representative biology for each module (Extended Data Fig. 3-4). Modules associated with cognition and disease status were identified from this SE2 network analysis. Three modules (M2, M5, M8) were associated significantly with disease status and AD neuropathology. M2 eigenprotein abundance was reduced in AD and AsymAD cases compared with controls. M2 GO terms related to mitochondrial functions, including hub proteins DLAT, ATP5PF, ATP5F1B, PDHB, TIMM13, and PHB2 (Fig. 3*B*). Decreased mitochondria proteins leads to dysregulated energy metabolism which is consistent with previous findings in AD (Stauch et al., 2014; Johnson et al., 2022; Walker et al., 2023; D’Alessandro et al., 2025). M5 eigenprotein abundance was increased in AD and AsymAD cases compared with controls. M5 was enriched with GO terms for DNA repair and contained hub proteins PRDX1, PDS5B, DHX15, MACROD1, PLEC, and HEPACAM (Fig. 3*C*). Elevated levels of M5 proteins reflect previous studies highlighting a link between impaired DNA repair and AD (Lin et al., 2020; Walker et al., 2023). M8 eigenprotein abundance was increased in AD and AsymAD cases compared with controls. M8 GO analysis revealed proteins associated with proteostasis, containing hub proteins NME7, UBR1, USP5, CUL5, PKM, and XPO1 (Fig. 3*D*). Increased M8 proteins observed in AD aligns with prior findings on proteostasis dysregulation in AD (Zhang et al., 2018; Barmaki et al., 2023). Notably, SE2 and WGCNA identified similar protein modules. We assessed hub protein overlap between each of the nine SE2 modules and their best, representative matching WGCNA module. The number of shared hub proteins was identified within each corresponding module pair as follows: SE2 M2 and WGCNA M10 shared two hub proteins, SE2 M8 and WGCNA M2 shared four hub proteins, SE2 M5 and WGCNA M6 shared two hub proteins, SE2 M7 and WGCNA M9 shared three hub proteins, SE2 M6 and WGCNA M5 shared two hub proteins, and SE2 M9 and WGCNA M8 shared two hub proteins. In total, 15 hub proteins overlapped between SE2 and WGCNA modules. Mitochondria-related modules from both networks (WGCNA M10 and SE2 M2) included DLAT and ATP5F1B as hub proteins (Figs. 2*E*, 3*B*). WGCNA M6 and SE2 M5 GO terms were associated with DNA repair and contained overlapping hub proteins MACROD1 and DHX15 (Figs. 2*B*, 3*C*). Overall, in comparison with WGCNA, SE2 network analysis yielded several parallel protein modules that are relevant to AD.

10.1523/ENEURO.0468-25.2026.f3-1 Figure 3-1 **Partition Comparison Heatmaps.** Partition Comparisons were performed to assess the similarity between WGCNA and SE2 protein clusters. Download Figure 3-1, ZIP file.

10.1523/ENEURO.0468-25.2026.f3-2 Figure 3-2 **Partition Comparison ARI and NMI Values.** Partition comparison analysis calculated the similarity in network clustering between WGCNA and SE2. Download Figure 3-2, XLS file.

10.1523/ENEURO.0468-25.2026.f3-3 Figure 3-3 S**E**2 **Module Assignments.** List of proteins assigned to each SE2 module. Download Figure 3-3, XLS file.

**Figure 4.**
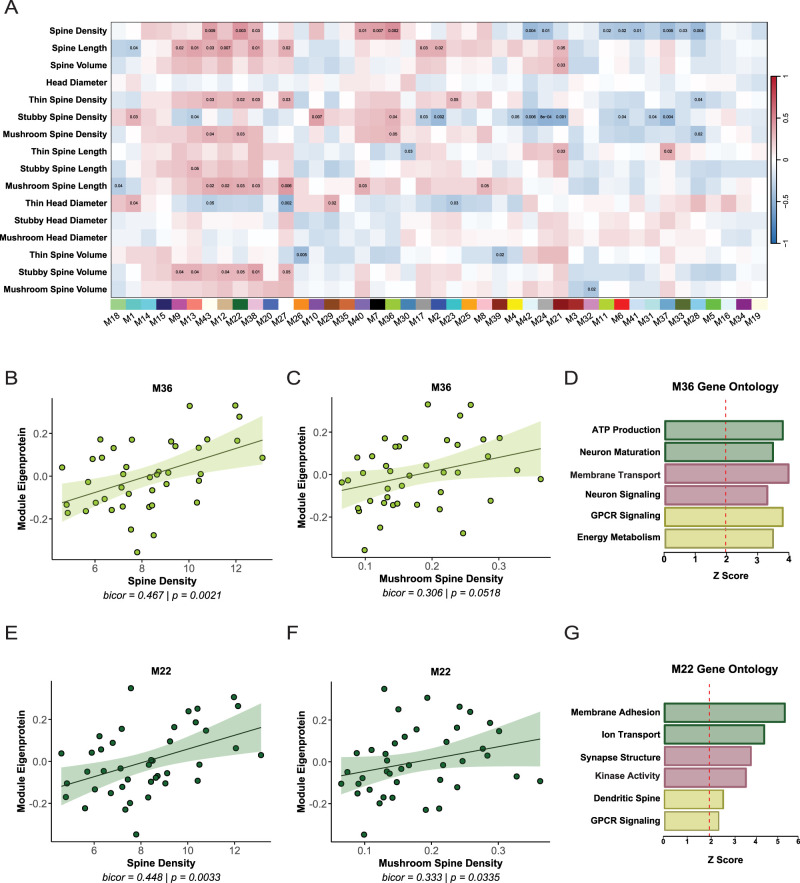
WGCNA network integration of dendritic spine density and morphology identifies synapse modules. ***A***, Biweight midcorrelations (bicor) between module eigenprotein expression and dendritic spine measurements. Red indicates positive correlations and blue indicates negative correlations, with color intensity reflecting correlation magnitude. Uncorrected *p* values are shown for correlations with *p* < 0.05. ***B***, Module 36 (M36) eigenprotein abundance is correlated positively with dendritic spine density. ***C***, M36 eigenprotein abundance is correlated positively with mushroom spine density. ***D***, Top GO terms for M36. ***E***, Module 22 (M22) eigenprotein abundance is correlated positively with dendritic spine density. ***F***, M22 eigenprotein abundance is correlated positively with mushroom spine density. Line of best fit was determined using a linear model, and the confidence interval is shaded around the line. ***B***, ***C***, ***E***, ***F***, Each point represents one case (*N* = 41). ***G***, Top GO terms for M22. *Z*-score above 1.96 was considered significant (*p* < 0.05).

10.1523/ENEURO.0468-25.2026.f3-4 Figure 3-4 S**E**2 **GO terms.** Gene ontology (GO) analysis was performed to gain insight into the biological meaning of each protein network module. Enrichment for a given ontology is shown by z score. Download Figure 3-4, ZIP file.

### WGCNA network integration of dendritic spine density and morphology identifies synapse module

WGCNA network integration of dendritic spine density and morphology measurements was conducted to determine protein modules that may be involved in spine biology and related to AD. Module eigenproteins were correlated with dendritic spine measurements from each case, using biweight midcorrelation. Several modules were correlated with spine density, including M43, M22, M38, M40, M7, M36, M42, M24, M11, M6, M41, M33, M37, and M28 (Fig. 4*A*). M36 and M22 were associated significantly with multiple dendritic spine traits, including overall spine density and mushroom spine density (Fig. 4*B*,*C*,*E*,*F*). M36 GO analysis revealed terms related to energy metabolism and neuron signaling (Fig. 4*D*). M22 GO analysis revealed terms related to synapse structure, ion transport, and dendritic spines (Fig. 4*G*). M22 and M36 eigenprotein abundances were reduced in AD cases (Fig. 2*F*,*G*); therefore, the positive correlation between spine density and M36 and M22 suggests that loss or dysregulation of these proteins contribute to structural vulnerability of spines in AD.

### SE2 network integration of dendritic spine density and morphology identifies synapse module

**Figure 5.**
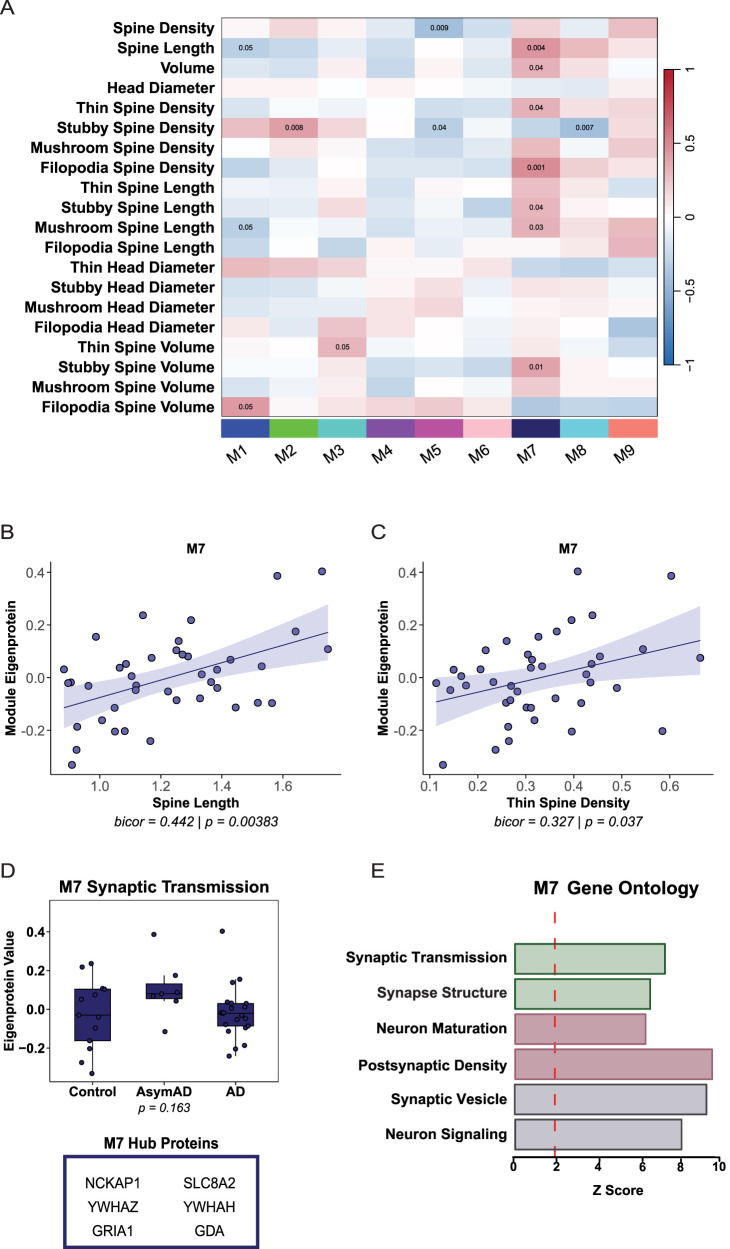
SE2 network integration of dendritic spine density and morphology identifies synapse module. ***A***, Biweight midcorrelations (bicor) between module eigenprotein abundance and dendritic spine measurements. Red indicates positive correlations and blue indicates negative correlations, with color intensity reflecting correlation magnitude. Uncorrected *p* values are shown for correlations with *p* < 0.05. ***B***, Module 7 (M7) eigenprotein abundance is associated positively with dendritic spine length. Line of best fit was determined using a linear model, and the confidence interval is shaded around the line. ***B***, ***C***, Each point represents one case (*N* = 41). ***C***, M7 eigenprotein abundance is associated positively with thin spine density. ***D***, M7 eigenprotein abundance is not differentially expressed between groups. ***E***, Top GO terms for M7. *Z*-score above 1.96 was considered significant (*p* < 0.05).

As an orthogonal approach, the dendritic spine density and morphology data was examined using SE2. SE2 module eigenproteins were correlated with dendritic spine measurements, using biweight midcorrelation. M1, M5, and M7 were correlated with two or more spine traits, including spine volume, length, or density (Fig. 5*A*). M7 was correlated significantly and positively with multiple dendritic spine traits, including spine length and thin spine density (Fig. 5*B*,*C*). M7 eigenprotein abundance was not differentially expressed between cases, and hub proteins included NCKAP1, YWHAZ, GRIA1, SLC8A2, YWHAH, and GDA (Fig. 5*D*). GO analysis revealed that M7 is linked to synapse structure, synaptic vesicle biology, postsynaptic density, and synaptic transmission (Fig. 5*E*).

### MDK, NTN1, and SMOC1 are associated with AD neuropathology and cognitive scores

**Figure 6.**
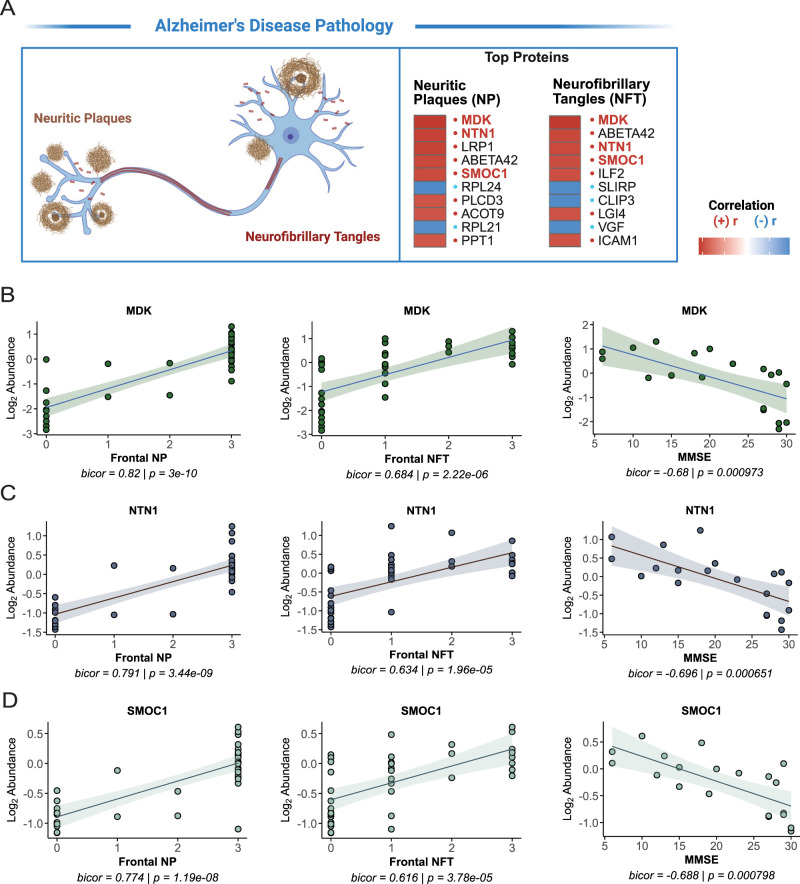
MDK, NTN1, and SMOC1 are associated with AD neuropathology and cognitive scores. TMT-MS quantified protein abundances were correlated with neuropathology using Pearson’s correlation. ***A***, Top proteins correlated with neurofibrillary tangles (NFT) and neuritic plaques (NP) are listed. Red indicates positive correlations and blue indicates negative correlations. Multiple comparisons were accounted for by FDR correction with a threshold of 0.10, and adjusted *q* values < 0.05. ***B***, MDK normalized abundance correlated with frontal NP, frontal NFT, and MMSE score. Line of best fit was determined using a linear model, and the confidence interval is shaded around the line. ***C***, NTN1 normalized abundance correlated with frontal NP, frontal NFT, and MMSE score. ***D***, SMOC1 normalized abundance correlated with frontal NP, frontal NFT, and MMSE score. Each point represents one case (*N* = 41). DP, NP, and NFTs are ordinal variables that represent neuropathology burden as follows: 0 = none, 1 = sparse, 2 = moderate, and 3 = frequent. See Extended Data Figure 6-1 for more details.

As a complementary approach to network-level analyses, we assessed the correlations between individual protein abundances and neuropathological scores. Normalized TMT-MS quantified protein abundances were correlated with AD neuropathology and cognitive scores. Protein abundances were Log2 transformed and normalized as a ratio dividing by the central tendency of pooled standards (GIS) and median centered. Highly correlated proteins are listed for neuritic plaques (NP) and neurofibrillary tangles (NFT; Fig. 6*A*, Extended Data Fig. 6-1). Among the top proteins, MDK, NTN1, and SMOC1 were associated positively with both NP and NFT scores. MDK, NTN1, and SMOC1 exhibited the same association pattern, with normalized abundances correlated positively with NP and NFT scores and inversely with MMSE scores (Fig. 6*B–D*). The results are highly consistent with past large-scale proteomic studies of the frontal cortex in AD that also identified MDK, NTN1, and SMOC1 and linked the proteins to AD neuropathology (Seyfried et al., 2017; Bai et al., 2020; Johnson et al., 2022). This further supports that our TMT-MS study herein is representative of the AD proteome from DLPFC.

10.1523/ENEURO.0468-25.2026.f6-1 Figure 6-1 A**D Neuropathology and Dendritic Spine Correlation Table.** Pearson correlation and FDR-adjusted q-values between normalized protein abundance data and neuropathology data and dendritic spine measurements. Download Figure 6-1, XLS file.

### NRN1 is a synaptic protein associated with AD

**Figure 7.**
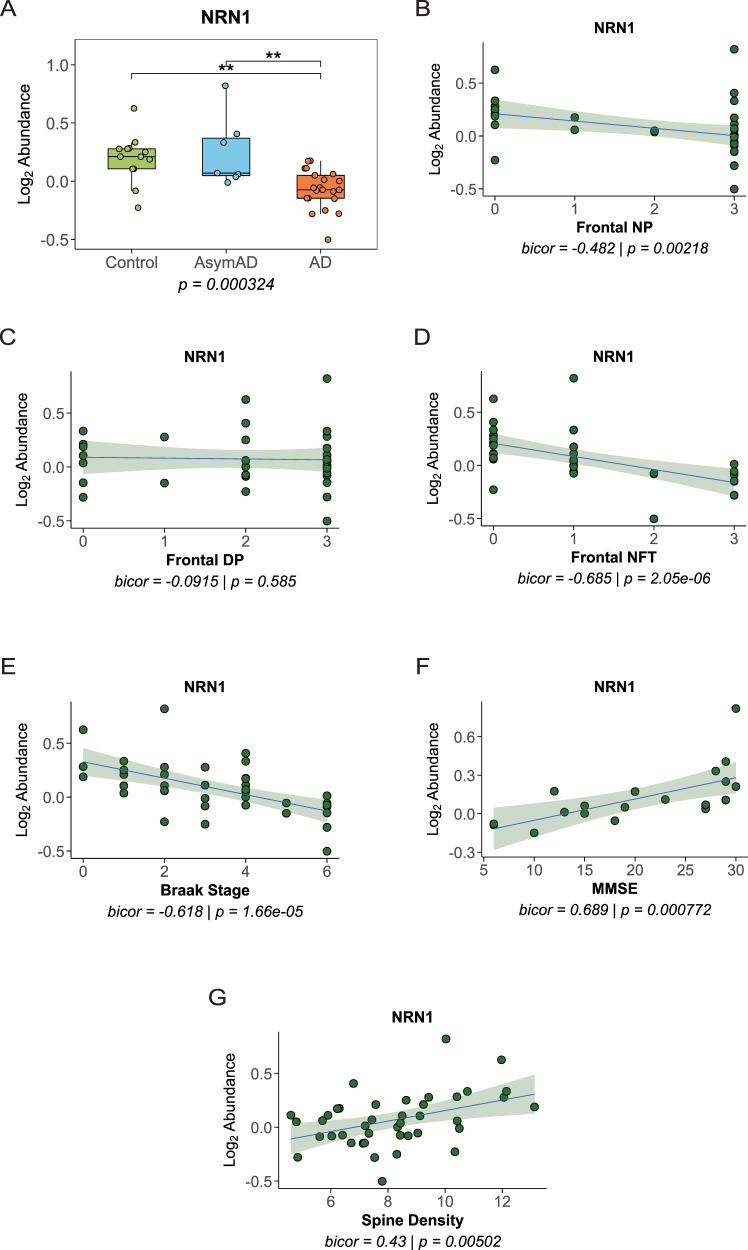
NRN1 is a synaptic protein associated with AD. ***A***, NRN1 protein abundance was comparable in AsymAD and controls but decreased in AD. One-way ANOVA (*F*_(2,38)_ = 10.00, *p* = 0.00032) with Tukey's multiple comparisons: controls versus AD, **p = 0.0019; AsymAD versus AD,**p = 0.0031. ***B***, NRN1 correlated inversely with frontal neuritic plaques (NP), using midweight bicorrelation. Line of best fit was determined using a linear model, and the confidence interval is shaded around the line. ***C***, NRN1 was not correlated with frontal diffuse plaques (DP). ***D***, NRN1 correlated inversely with frontal neurofibrillary tangles (NFT). DP, NP, and NFTs are ordinal variables that represent neuropathology burden as follows: 0 = none, 1 = sparse, 2 = moderate, and 3 = frequent. ***E***, NRN1 correlated positively with MMSE. ***F***, NRN1 correlated inversely with Braak stage. ***G***, NRN1 correlated positively with spine density. Each point represents one case (*N* = 41). See Extended Data Figure 2-3 for more details.

Next, we conducted tests to analyze the synaptic protein neuritin 1 (NRN1) as it relates to AD neuropathology and cognitive scores. NRN1 is a secreted neuropeptide and functions to promote synapse maturation and dendritic spine generation (Fujino et al., 2011; Subramanian et al., 2019). NRN1 was identified previously in several large-scale multi-omic studies as a protein associated with cognitive resilience in AD by supporting synaptic preservation and maintenance (Yu et al., 2020; Hurst et al., 2023; Zammit et al., 2024). Consistent with that, NRN1 was identified as a hub protein in WGCNA M36 with GO terms suggesting relevance to neuron signaling (Fig. 2*G*, Extended Data Fig. 2-3). NRN1 protein abundances were Log2 transformed and normalized as a ratio dividing by the central tendency of pooled standards (GIS) and median centered. Consistent with the previous reports above, NRN1 normalized protein abundance was comparable in AsymAD and controls but decreased significantly in AD cases (Fig. 7*A*). Furthermore, biweight midcorrelation was performed to assess protein correlations with AD neuropathology, which demonstrated that NRN1 was correlated inversely with frontal neuritic plaques, but not frontal diffuse plaques (Fig. 7*B*,*C*). NRN1 was correlated inversely with frontal NFTs and Braak score but positively with MMSE score and overall spine density (Fig. 7*D–G*). The identification and analysis of NRN1 in this dataset is highly consistent with past findings and further supports the putative importance of NRN1 as a key synaptic protein in AD (Hurst et al., 2023; Pugh et al., 2026).

### Protein associations with dendritic spine density and morphology

**Figure 8.**
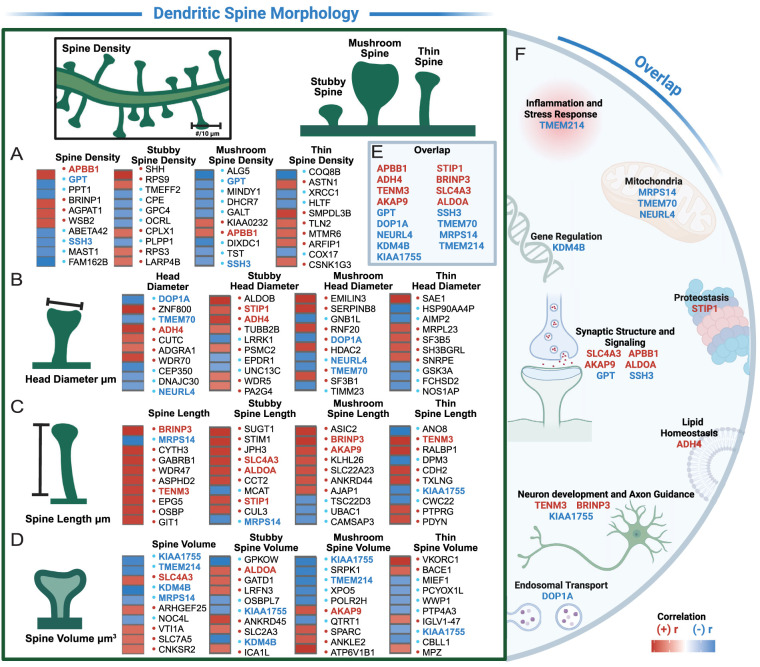
Protein associations with dendritic spine density and morphology. Normalized TMT-MS quantified individual protein abundances were associated with dendritic spine density and morphology using Pearson’s correlation and FDR-adjusted significant *q* values (BH FDR < 0.10 and *q* < 0.05; Extended Data Fig. 6-1). Positive correlations are shown in red and negative correlations in blue. Bolded proteins indicate overlap across spine traits. ***A***, Top proteins correlated with spine density. ***B***, Top proteins correlated with spine head diameter. ***C***, Top proteins correlated with spine length. ***D***, Top proteins correlated with spine volume. ***E***, Proteins with overlap across spine measurements. ***F***, Biological relevance of overlapping proteins. See Extended Data Figure 6-1 for more details.

To identify proteins associated with dendritic spine biology, we performed correlations between individual protein abundances and dendritic spine density and morphology across all cases (irrespective of disease state). Pearson’s correlation analyses were performed between individual protein abundance and dendritic spine traits. Multiple testing correction was applied using both the BH method and Storey's *q* value estimation to control the FDR. Normalized TMT-MS quantified protein abundances were correlated with spine density, spine head diameter, spine length, and spine volume and were further categorized by spine classes (stubby, mushroom, and thin). For each protein, we associated its abundance with dendritic spine measurements, while accounting for batch effects and PMI. The 10 proteins with the lowest *q* values are listed for each spine trait (Fig. 8*A–D*; Extended Data Fig. 6-1). Proteins that appeared more than once across the spine measurements were highlighted as an overlapping protein (Fig. 8*E*). Each overlapping protein was then binned into functional themes based on the protein's known/predicted cellular role (Fig. 8*F*). This analysis identified numerous proteins linked to cellular processes, including mitochondria, proteostasis, and synaptic transmission, that likely impact spine density and morphology in AD.

## Discussion

In the present study, we utilized two complementary network analysis approaches that integrated multi-scale data, including MS-based proteomics and dendritic spine morphometry, to identify proteins linked to dendritic spine alterations in AD. Proteomic correlation networks have been applied successfully to study proteome-wide changes across disease states and identify candidate biomarkers and therapeutic targets in AD (Langfelder and Horvath, 2008; Mostafavi et al., 2018). Herein, WGCNA and SE2 yielded network-distinct and overlapping protein modules that were differentially abundant in AD cases compared with controls and AsymAD. Notably, the mitochondria and DNA repair modules shared hub proteins across WGCNA and SE2 networks, emphasizing how these neurobiological themes are altered in AD.

By integrating proteomic data with spine morphology measurements, we identified spine-relevant proteins involved in cellular metabolism, inflammation, protein homeostasis, and synaptic signaling. TMEM214, linked to cellular stress pathways, exhibited negative correlation with overall spine volume and mushroom spine volume. TMEM214 is localized to the outer membrane of the endoplasmic reticulum and functions as a critical mediator of stress-induced apoptosis (Li et al., 2013). While TMEM214 has not been linked directly to AD, neuroinflammation is closely associated with AD progression, and proteins that regulate cellular stress responses may contribute to synaptic dysfunction in AD (Müller and Di Benedetto, 2025). We also identified DOP1A, a protein linked to endosomal transport functions, that was negatively correlated with overall spine head diameter and mushroom spine head diameter. The endosomal transport system is critical for maintaining synaptic integrity by regulating transmembrane receptor recycling, vesicle traffic, and membrane turnover. Endosomal traffic is dysregulated early in AD and is hypothesized to disrupt synapse homeostasis (Pensalfini et al., 2020). Although DOP1A has not been linked directly to AD, proper endosomal transport is essential for synaptic maintenance. Dysregulation of DOP1A may contribute to synaptic vulnerability in AD (Pensalfini et al., 2020). Several proteins were also linked with synaptic structure and signaling, including SLC4A3, APBB1, AKAP9, ALDOA, GPT, and SSH3. APBB1 was correlated positively with spine density and mushroom spine density and has been linked to AD. APBB1 interacts with amyloid precursor protein and plays a central role in actin regulation during synaptic plasticity (Augustin and Kins, 2021). Additionally, AKAP9 was correlated positively with mushroom spine length and mushroom spine volume. Genetic studies identified AKAP9 coding SNPs associated with AD risk in certain populations (Logue et al., 2014). AKAP9 functions as a scaffold protein to regulate activity of kinases involved in synaptic structure and intracellular signaling (Sanderson and Dell’Acqua, 2011; Augustin and Kins, 2021).

NRN1 was initially identified as a highly promising therapeutic target for AD from the Religious Orders Study and the Rush Memory and Aging Project (Yu et al., 2020; Hurst et al., 2023; Pugh et al., 2026). However, to provide the confidence necessary for massive therapeutic investment, validation and reproducibility of targets is required to extend across multiple human cohorts. NRN1 lacked this but remained a notable target to support synapses in AD. NRN1, also known as candidate plasticity gene 15 (CPG15), is a synaptic activity-regulated gene that modulates the formation of axonal arbors and dendritic branching (Naeve et al., 1997; Fujino et al., 2011; An et al., 2014; Subramanian et al., 2019). Parallel to studies in human aging populations, NRN1 was shown to protect synapses, both structurally and physiologically, from Aβ in animal and cellular models of AD (An et al., 2014; Hurst et al., 2023). The therapeutic value of NRN1 continued to soar when NRN1 was identified as a critical synaptic protein that heavily influenced functional connectivity emanating from the inferior temporal gyrus in aging individuals with AD neuropathology (Ng et al., 2024). Yet despite these findings, NRN1's associations with human cognition, neuropathology, and synaptic biology in AD stemmed mostly from ROSMAP data. Results herein indicate that NRN1 protein is reduced in AD compared with controls and AsymAD brains from the Emory University Alzheimer's Disease Research Center. Moreover, NRN1 was associated positively with cognition and spine density, as well as negatively correlated with neuropathology, in these individuals. Hence, our findings on NRN1 reproduce and validate results from the ROSMAP cohort and support the impetus for translational research on NRN1 (Yu et al., 2020; Hurst et al., 2023; Pugh et al., 2026). Notably, NRN1 was correlated inversely with neuritic plaques but not diffuse plaques in this analysis (Fig. 7*B*,*C*). Diffuse plaques can be associated with normal aging without cognitive impairment, whereas neuritic plaques are linked with AD progression and cognitive decline (Haroutunian et al., 1998; Malek-Ahmadi et al., 2016; Tsering and Prokop, 2024). These findings are highly consistent with NRN1 protein levels remaining normal in controls and AsymAD cases but reduced significantly in AD.

Our findings suggest several considerations when interpreting proteomic network data. One factor to consider is the brain region specificity of protein abundances and dendritic spine measurements. Assessing protein levels and spine morphology across additional brain regions within the same individuals could provide deeper insight into the extent to which protein abundances contribute similarly or differentially to structural and functional features across brain regions. Moreover, bulk homogenate proteomic measurements inherently feature limitations related to assigning protein abundance changes to specific cellular compartments, like synapses, or cell types. For instance, while we discuss proteins in the context of dendritic spines, these proteins may originate from nonsynaptic areas of the neuron or non-neuronal cells.

WGCNA module structure is influenced by several parameters, including soft-threshold power, dynamic tree cutting, and merge cut height. These parameters determine WGCNA network topology and how modules are clustered (Zhang and Horvath, 2005; Horvath and Dong, 2008; Langfelder et al., 2008; Langfelder and Horvath, 2008). In contrast, SE2 applies an iterative consensus-based clustering method and does not rely on the same module structure as WGCNA. SE2 module clustering is determined by dynamic node assignment and optional subclustering as well as minimum cluster size constraints to refine module granularity (Gaiteri et al., 2023). Because WGCNA and SE2 have different definitions of network organization and module structure, which are shaped by their unique parameter settings and cluster detection frameworks, fully overlapping hub proteins are not expected but some hub proteins show up in both approaches.

In conclusion, this rigorous multi-network approach, integrating dendritic spine measurements with proteomic analysis, enables the identification of proteins relevant to spines and synapses in AD. Additional future studies will require experimental validation to provide deeper understanding of the mechanisms underlying these cellular and molecular changes in AD.

## Synthesis

Reviewing Editor: Laura Volpicelli-Daley, The University of Alabama at Birmingham Decisions are customarily a result of the Reviewing Editor and the peer reviewers coming together and discussing their recommendations until a consensus is reached. When revisions are invited, a fact-based synthesis statement explaining their decision and outlining what is needed to prepare a revision will be listed below. The following reviewer(s) agreed to reveal their identity: NONE.

Overall, both reviewers felt the proteomics and spine analyses in this study were well done. Even though the results are largely confirmatory, the data is strong and useful for the field because it strengthens identified networks and proteins.

The manuscript would be further strengthened with the following recommended additions and changes:

Please provide primary data related to dendritic spine reconstructions in main figures. At a minimum, summary plots describing the relationship between each of the spine measures and individual AD scores would be helpful in evaluating the dataset.

For figure 2, please compare protein abundance data between groups to show that increase or decrease in eigenprotein value is associated with increase or decrease in hub protein abundance and fraction of hub proteins whose fold changes match module direction.

Please report the fraction of overlapping hub proteins between WGCNA module and best matching SE2 module to support statement that WGCNA settings that produce larger modules look are similar to SE2.

Present protein correlations as heat maps. This will allow the reader to more easily evaluate the strength of each correlation. While this information is found in the supplemental table, it requires some digging to find each of the proteins highlighted in the main figure.

Define X values for DP, NP and NFT.

In the discussion section:

Further rationale for highlighting NRN1.

Discuss why some hub proteins are detected by both approaches but others or not.
