# Supplementary material for: A Multi-Network Approach Identifies Proteins Related to Dendritic Spines in Alzheimer’s Disease
Source: eNeuro. 2026 Apr 10;13(4):ENEURO.0468-25.2026. doi: 10.1523/ENEURO.0468-25.2026 (PMC13095402; doi:10.1523/ENEURO.0468-25.2026)

Extended Data Figure 1-2. Dendritic Spine Measurements Across Conditions.
One−way ANOVA with Tukey post hoc comparisons; Grubbs' test used for outlier detection.

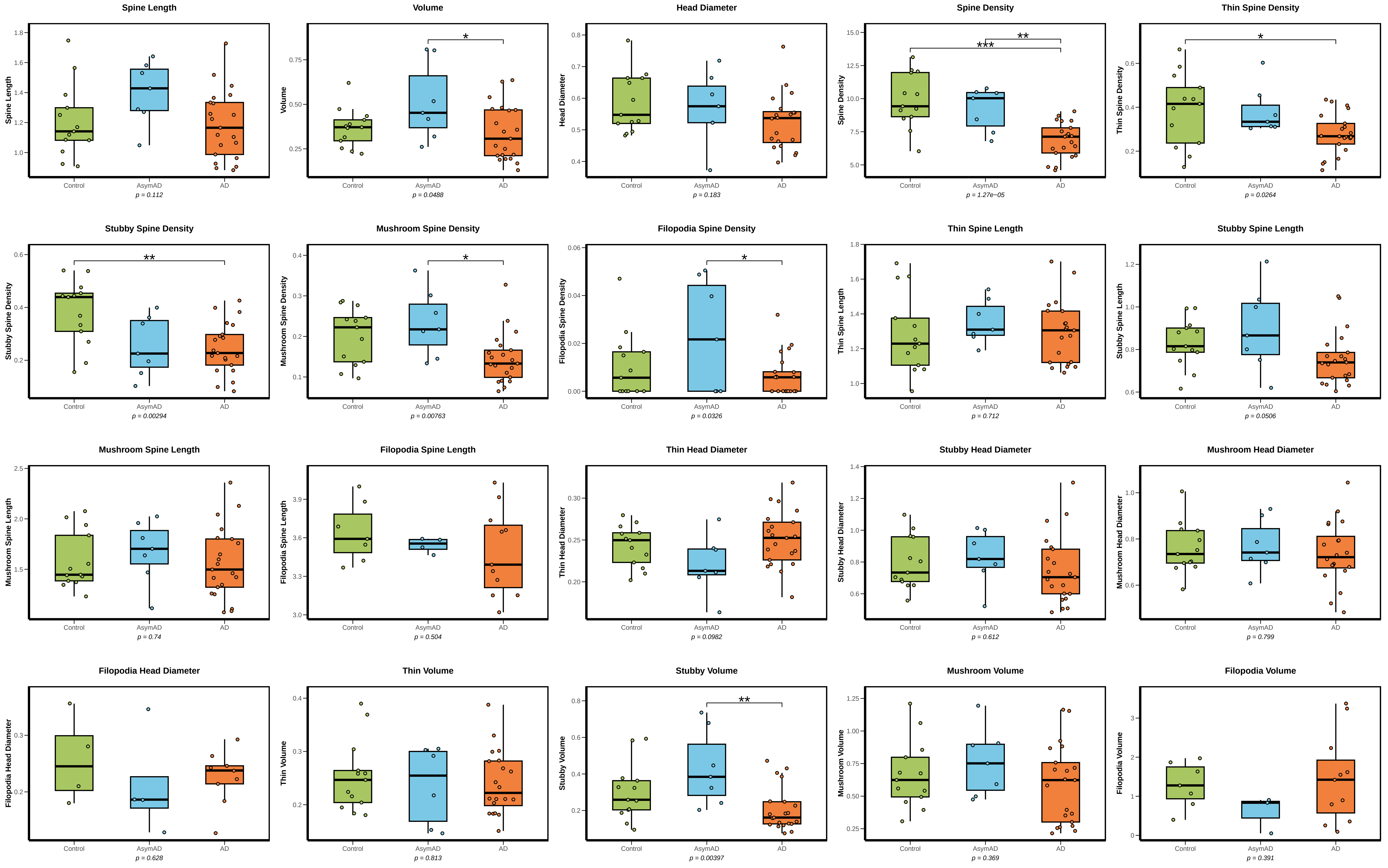

Supplement: Figure 1-2 — Dendritic Spine Measurements Across Conditions. Box plots of dendritic spine measurements across Control, AsymAD, and AD groups. One−way ANOVA with Tukey post hoc comparisons, and Grubbs’ test used for outlier detection. Download Figure 1-2, ZIP file. [file eneuro-13-ENEURO.0468-25.2026-s004.zip › Extended Data Figure 1-2.pdf]
