# Supplementary material for: A Multi-Network Approach Identifies Proteins Related to Dendritic Spines in Alzheimer’s Disease
Source: eNeuro. 2026 Apr 10;13(4):ENEURO.0468-25.2026. doi: 10.1523/ENEURO.0468-25.2026 (PMC13095402; doi:10.1523/ENEURO.0468-25.2026)
Supplement: Figure 2-2 — WGCNA Hub Protein Plots. Box plots of WGCNA hub proteins, each colored with respective module color. Log2 abundance values across Control, AsymAD, and AD groups. Download Figure 2-2, ZIP file. [file eneuro-13-ENEURO.0468-25.2026-s007.zip › Extended Data Figure 2-2.pdf]

Extended Data Figure 2-2. WGCNA Hub Protein Plots.

Each protein colored by module; Log2 abundance values across Control, AsymAD, and AD groups.

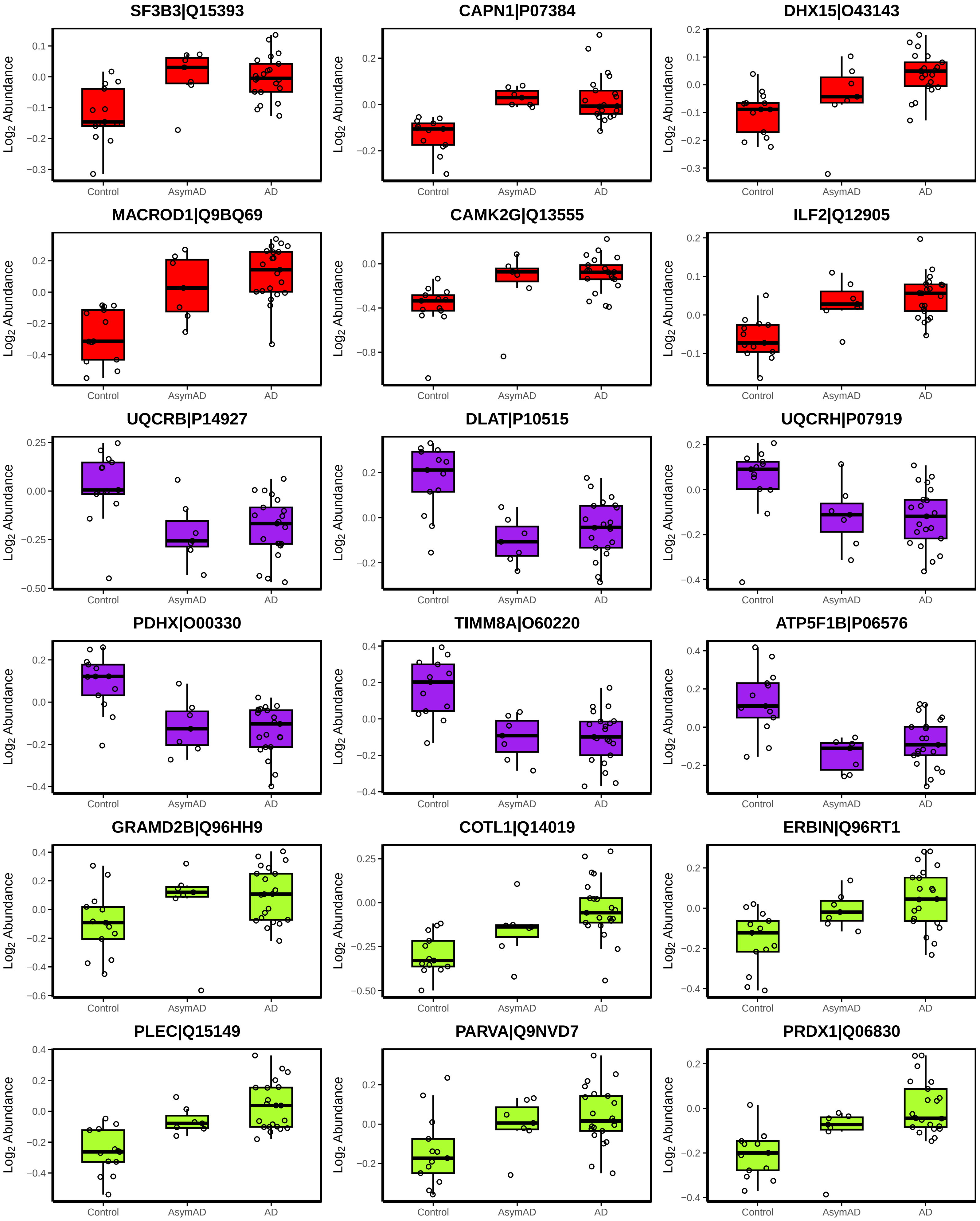

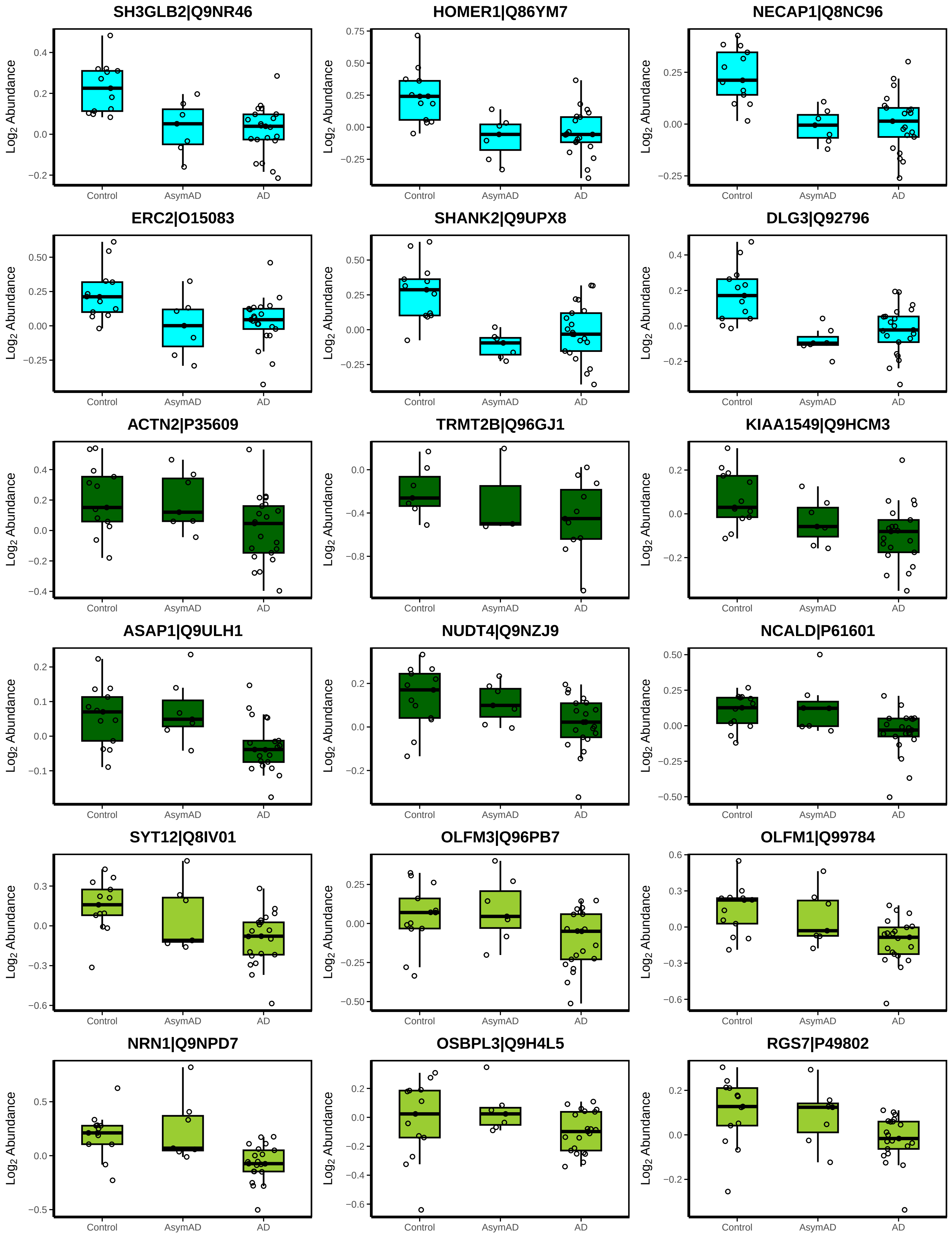

SH3GLB2|Q9NR46
HOMER1|Q86YM7
NECAP1|Q8NC96
ERC2|O15083
SHANK2|Q9UPX8
DLG3|Q92796
ACTN2|P35609
TRMT2B|Q96GJ1
KIAA1549|Q9HCM3
ASAP1|Q9ULH1
NUDT4|Q9NZJ9
NCALD|P61601
SYT12|Q8IV01
OLFM3|Q96PB7
OLFM1|Q99784
NRN1|Q9NPD7
OSBPL3|Q9H4L5
RGS7|P49802
Log2 Abundance
Control
AsymAD
AD
