# Supplementary material for: A Multi-Network Approach Identifies Proteins Related to Dendritic Spines in Alzheimer’s Disease
Source: eNeuro. 2026 Apr 10;13(4):ENEURO.0468-25.2026. doi: 10.1523/ENEURO.0468-25.2026 (PMC13095402; doi:10.1523/ENEURO.0468-25.2026)
Supplement: Figure 2-3 — WGCNA GO terms. Gene ontology (GO) analysis was performed to gain insight into the biological meaning of each protein network module. Enrichment for a given ontology is shown by z score. Download Figure 2-3, ZIP file. [file eneuro-13-ENEURO.0468-25.2026-s008.zip › Extended Data Figure 2-3.pdf]

Extended Data Figure 2-3. GO Analysis on WGCNA Network Modules. Gene ontology (GO) analysis was performed to gain insight into the biological meaning of each protein network module. Enrichment for a given ontology is shown by z score.

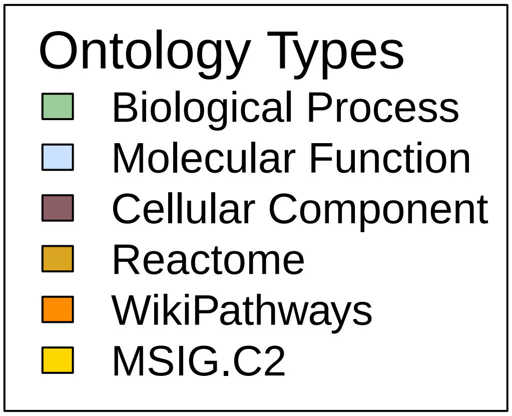

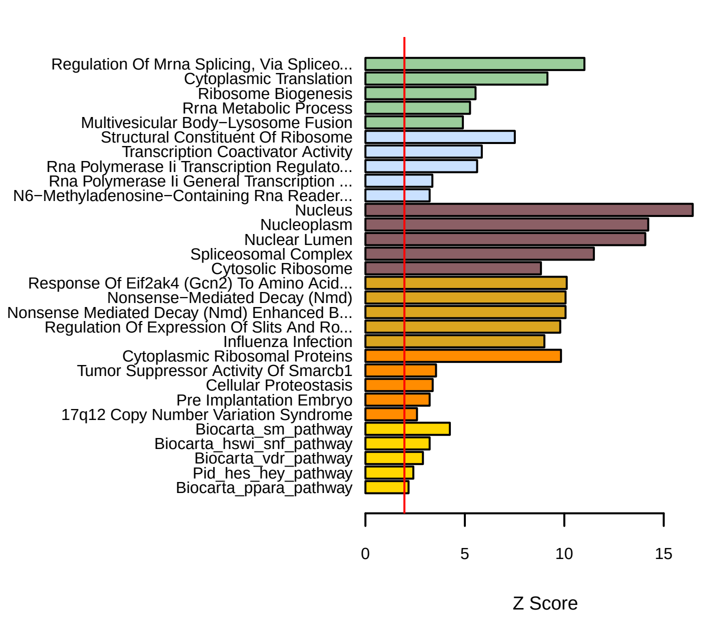

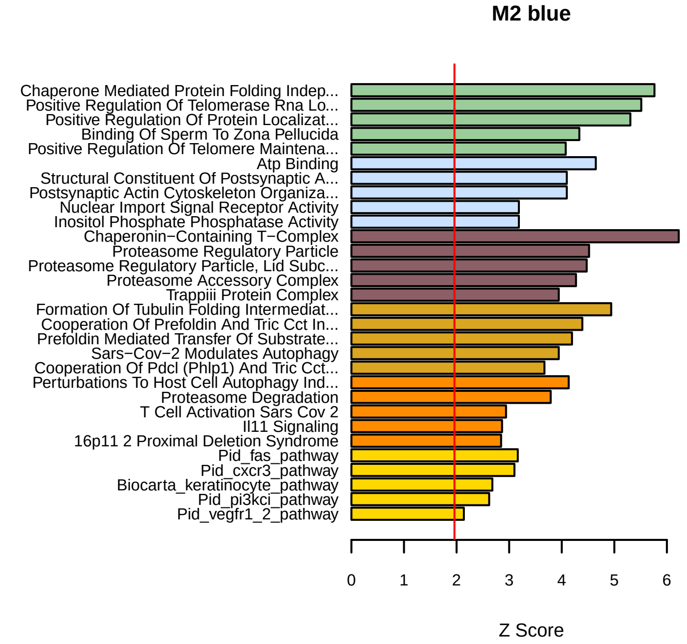

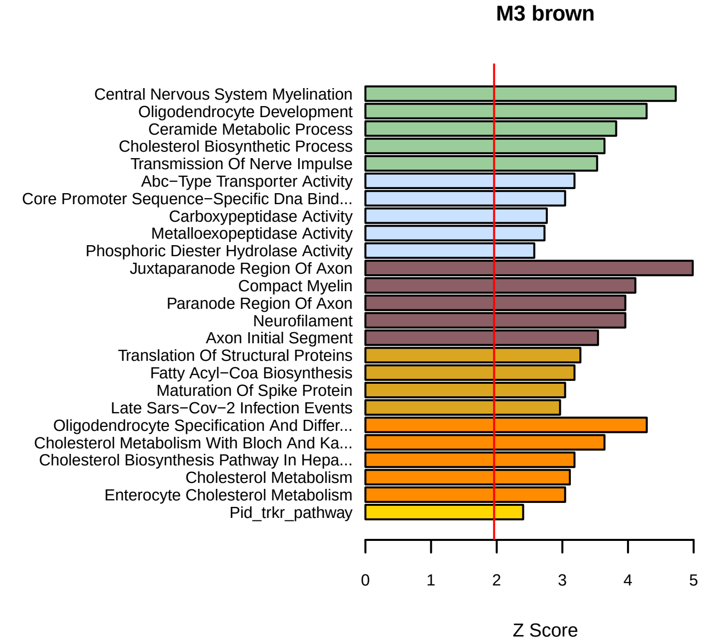

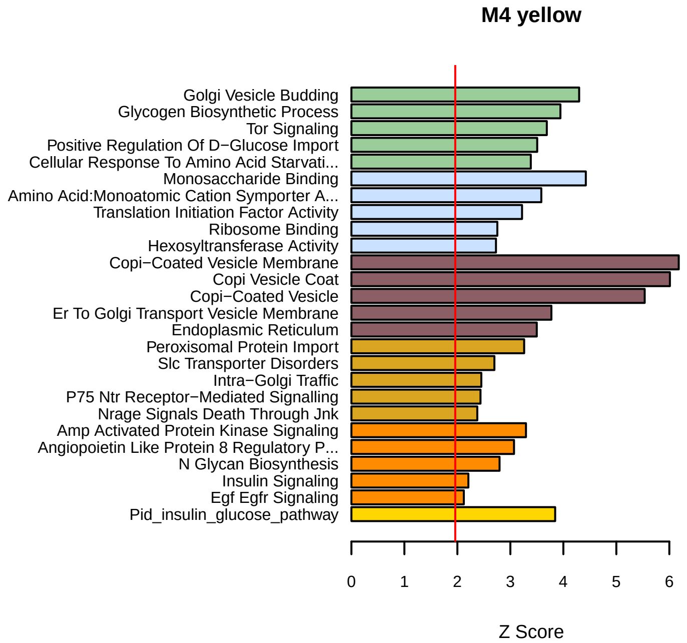

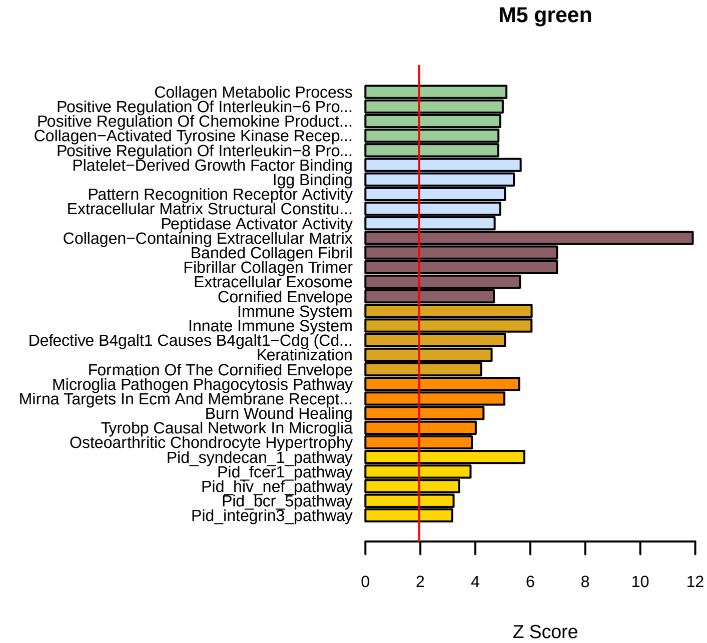

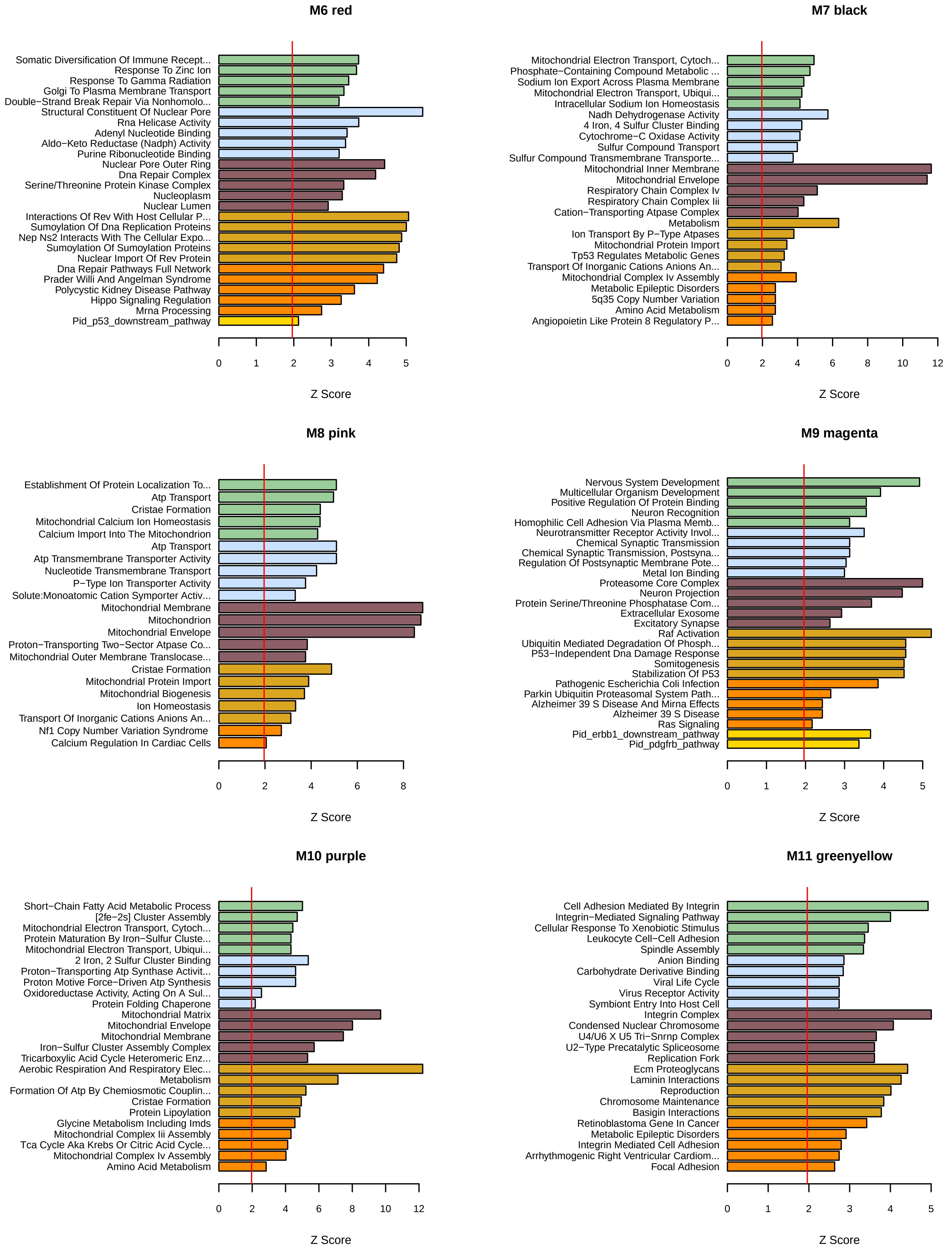
M6 red
Somatic Diversification Of Immune Recept...
Response To Zinc Ion
Response To Gamma Radiation
Golgi To Plasma Membrane Transport
Double−Strand Break Repair Via Nonhomolo...
Structural Constituent Of Nuclear Pore
Rna Helicase Activity
Adenyl Nucleotide Binding
Aldo−Keto Reductase (Nadph) Activity
Purine Ribonucleotide Binding
Nuclear Pore Outer Ring
Dna Repair Complex
Serine/Threonine Protein Kinase Complex
Nucleoplasm
Nuclear Lumen
Interactions Of Rev With Host Cellular P...
Sumoylation Of Dna Replication Proteins
Nep Ns2 Interacts With The Cellular Expo...
Sumoylation Of Sumoylation Proteins
Nuclear Import Of Rev Protein
Dna Repair Pathways Full Network
Prader Willi And Angelman Syndrome
Polycystic Kidney Disease Pathway
Hippo Signaling Regulation
Mrna Processing
Pid_p53_downstream_pathway
0 1 2 3 4 5
Z Score
M7 black
Mitochondrial Electron Transport, Cytoch...
Phosphate−Containing Compound Metabolic ...
Sodium Ion Export Across Plasma Membrane
Mitochondrial Electron Transport, Ubiqui...
Intracellular Sodium Ion Homeostasis
Nadh Dehydrogenase Activity
4 Iron, 4 Sulfur Cluster Binding
Cytochrome−C Oxidase Activity
Sulfur Compound Transport
Sulfur Compound Transmembrane Transporte...
Mitochondrial Inner Membrane
Mitochondrial Envelope
Respiratory Chain Complex Iv
Respiratory Chain Complex Iii
Cation−Transporting Atpase Complex
Metabolism
Ion Transport By P−Type Atpases
Mitochondrial Protein Import
Tp53 Regulates Metabolic Genes
Transport Of Inorganic Cations Anions An...
Mitochondrial Complex Iv Assembly
Metabolic Epileptic Disorders
5q35 Copy Number Variation
Amino Acid Metabolism
Angiopoietin Like Protein 8 Regulatory P...
0 2 4 6 8 10 12
Z Score
M8 pink
Establishment Of Protein Localization To...
Atp Transport
Cristae Formation
Mitochondrial Calcium Ion Homeostasis
Calcium Import Into The Mitochondrion
Atp Transport
Atp Transmembrane Transporter Activity
Nucleotide Transmembrane Transport
P−Type Ion Transporter Activity
Solute:Monoatomic Cation Symporter Activ...
Mitochondrial Membrane
Mitochondrion
Mitochondrial Envelope
Proton−Transporting Two−Sector Atpase Co...
Mitochondrial Outer Membrane Translocase...
Cristae Formation
Mitochondrial Protein Import
Mitochondrial Biogenesis
Ion Homeostasis
Transport Of Inorganic Cations Anions An...
Nf1 Copy Number Variation Syndrome
Calcium Regulation In Cardiac Cells
0 2 4 6 8
Z Score
M9 magenta
Nervous System Development
Multicellular Organism Development
Positive Regulation Of Protein Binding
Neuron Recognition
Homophilic Cell Adhesion Via Plasma Memb...
Neurotransmitter Receptor Activity Invol...
Chemical Synaptic Transmission
Chemical Synaptic Transmission, Postsyna...
Regulation Of Postsynaptic Membrane Pote...
Metal Ion Binding
Proteasome Core Complex
Neuron Projection
Protein Serine/Threonine Phosphatase Com...
Extracellular Exosome
Excitatory Synapse
Raf Activation
Ubiquitin Mediated Degradation Of Phosph...
P53−Independent Dna Damage Response
Somitogenesis
Stabilization Of P53
Pathogenic Escherichia Coli Infection
Parkin Ubiquitin Proteasomal System Path...
Alzheimer 39 S Disease And Mirna Effects
Alzheimer 39 S Disease
Ras Signaling
Pid_erbb1_downstream_pathway
Pid_pdgfrb_pathway
0 1 2 3 4 5
Z Score
M10 purple
Short−Chain Fatty Acid Metabolic Process
[2fe−2s] Cluster Assembly
Mitochondrial Electron Transport, Cytoch...
Protein Maturation By Iron−Sulfur Cluste...
Mitochondrial Electron Transport, Ubiqui...
2 Iron, 2 Sulfur Cluster Binding
Proton−Transporting Atp Synthase Activit...
Proton Motive Force−Driven Atp Synthesis
Oxidoreductase Activity, Acting On A Sul...
Protein Folding Chaperone
Mitochondrial Matrix
Mitochondrial Envelope
Mitochondrial Membrane
Iron−Sulfur Cluster Assembly Complex
Tricarboxylic Acid Cycle Heteromeric Enz...
Aerobic Respiration And Respiratory Elec...
Metabolism
Formation Of Atp By Chemiosmotic Couplin...
Cristae Formation
Protein Lipoylation
Glycine Metabolism Including Imds
Mitochondrial Complex Iii Assembly
Tca Cycle Aka Krebs Or Citric Acid Cycle...
Mitochondrial Complex Iv Assembly
Amino Acid Metabolism
0 2 4 6 8 10 12
Z Score
M11 greenyellow
Cell Adhesion Mediated By Integrin
Integrin−Mediated Signaling Pathway
Cellular Response To Xenobiotic Stimulus
Leukocyte Cell−Cell Adhesion
Spindle Assembly
Anion Binding
Carbohydrate Derivative Binding
Viral Life Cycle
Virus Receptor Activity
Symbiont Entry Into Host Cell
Integrin Complex
Condensed Nuclear Chromosome
U4/U6 X U5 Tri−Snrnp Complex
U2−Type Precatalytic Spliceosome
Replication Fork
Ecm Proteoglycans
Laminin Interactions
Reproduction
Chromosome Maintenance
Basigin Interactions
Retinoblastoma Gene In Cancer
Metabolic Epileptic Disorders
Integrin Mediated Cell Adhesion
Arrhythmogenic Right Ventricular Cardiom...
Focal Adhesion
0 1 2 3 4 5
Z Score

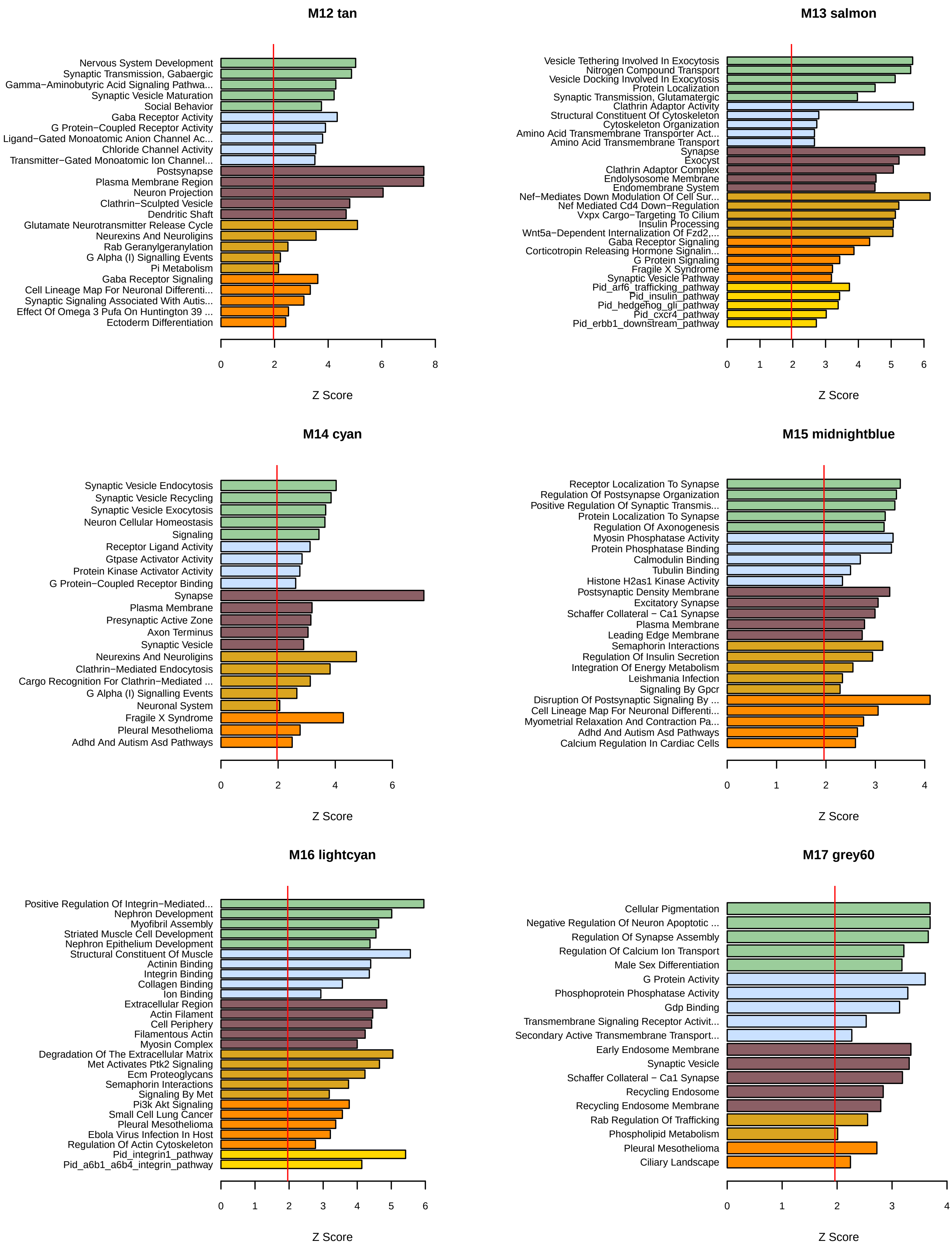
M12 tan
Nervous System Development
Synaptic Transmission, Gabaergic
Gamma−Aminobutyric Acid Signaling Pathwa...
Synaptic Vesicle Maturation
Social Behavior
Gaba Receptor Activity
G Protein−Coupled Receptor Activity
Ligand−Gated Monoatomic Anion Channel Ac...
Chloride Channel Activity
Transmitter−Gated Monoatomic Ion Channel...
Postsynapse
Plasma Membrane Region
Neuron Projection
Clathrin−Sculpted Vesicle
Dendritic Shaft
Glutamate Neurotransmitter Release Cycle
Neurexins And Neuroligins
Rab Geranylgeranylation
G Alpha (I) Signalling Events
Pi Metabolism
Gaba Receptor Signaling
Cell Lineage Map For Neuronal Differenti...
Synaptic Signaling Associated With Autis...
Effect Of Omega 3 Pufa On Huntington 39 ...
Ectoderm Differentiation
0 2 4 6 8
Z Score
M13 salmon
Vesicle Tethering Involved In Exocytosis
Nitrogen Compound Transport
Vesicle Docking Involved In Exocytosis
Protein Localization
Synaptic Transmission, Glutamatergic
Clathrin Adaptor Activity
Structural Constituent Of Cytoskeleton
Cytoskeleton Organization
Amino Acid Transmembrane Transporter Act...
Amino Acid Transmembrane Transport
Synapse
Exocyst
Clathrin Adaptor Complex
Endolysosome Membrane
Endomembrane System
Nef−Mediates Down Modulation Of Cell Sur...
Nef Mediated Cd4 Down−Regulation
Vxpx Cargo−Targeting To Cilium
Insulin Processing
Wnt5a−Dependent Internalization Of Fzd2,...
Gaba Receptor Signaling
Corticotropin Releasing Hormone Signalin...
G Protein Signaling
Fragile X Syndrome
Synaptic Vesicle Pathway
Pid_arf6_trafficking_pathway
Pid_insulin_pathway
Pid_hedgehog_gli_pathway
Pid_cxcr4_pathway
Pid_erbb1_downstream_pathway
0 1 2 3 4 5 6
Z Score
M14 cyan
Synaptic Vesicle Endocytosis
Synaptic Vesicle Recycling
Synaptic Vesicle Exocytosis
Neuron Cellular Homeostasis
Signaling
Receptor Ligand Activity
Gtpase Activator Activity
Protein Kinase Activator Activity
G Protein−Coupled Receptor Binding
Synapse
Plasma Membrane
Presynaptic Active Zone
Axon Terminus
Synaptic Vesicle
Neurexins And Neuroligins
Clathrin−Mediated Endocytosis
Cargo Recognition For Clathrin−Mediated ...
G Alpha (I) Signalling Events
Neuronal System
Fragile X Syndrome
Pleural Mesothelioma
Adhd And Autism Asd Pathways
0 2 4 6
Z Score
M15 midnightblue
Receptor Localization To Synapse
Regulation Of Postsynapse Organization
Positive Regulation Of Synaptic Transmis...
Protein Localization To Synapse
Regulation Of Axonogenesis
Myosin Phosphatase Activity
Protein Phosphatase Binding
Calmodulin Binding
Tubulin Binding
Histone H2as1 Kinase Activity
Postsynaptic Density Membrane
Excitatory Synapse
Schaffer Collateral − Ca1 Synapse
Plasma Membrane
Leading Edge Membrane
Semaphorin Interactions
Regulation Of Insulin Secretion
Integration Of Energy Metabolism
Leishmania Infection
Signaling By Gpcr
Disruption Of Postsynaptic Signaling By ...
Cell Lineage Map For Neuronal Differenti...
Myometrial Relaxation And Contraction Pa...
Adhd And Autism Asd Pathways
Calcium Regulation In Cardiac Cells
0 1 2 3 4
Z Score
M16 lightcyan
Positive Regulation Of Integrin−Mediated...
Nephron Development
Myofibril Assembly
Striated Muscle Cell Development
Nephron Epithelium Development
Structural Constituent Of Muscle
Actinin Binding
Integrin Binding
Collagen Binding
Ion Binding
Extracellular Region
Actin Filament
Cell Periphery
Filamentous Actin
Myosin Complex
Degradation Of The Extracellular Matrix
Met Activates Ptk2 Signaling
Ecm Proteoglycans
Semaphorin Interactions
Signaling By Met
Pi3k Akt Signaling
Small Cell Lung Cancer
Pleural Mesothelioma
Ebola Virus Infection In Host
Regulation Of Actin Cytoskeleton
Pid_integrin1_pathway
Pid_a6b1_a6b4_integrin_pathway
0 1 2 3 4 5 6
Z Score
M17 grey60
Cellular Pigmentation
Negative Regulation Of Neuron Apoptotic ...
Regulation Of Synapse Assembly
Regulation Of Calcium Ion Transport
Male Sex Differentiation
G Protein Activity
Phosphoprotein Phosphatase Activity
Gdp Binding
Transmembrane Signaling Receptor Activit...
Secondary Active Transmembrane Transport...
Early Endosome Membrane
Synaptic Vesicle
Schaffer Collateral − Ca1 Synapse
Recycling Endosome
Recycling Endosome Membrane
Rab Regulation Of Trafficking
Phospholipid Metabolism
Pleural Mesothelioma
Ciliary Landscape
0 1 2 3 4
Z Score

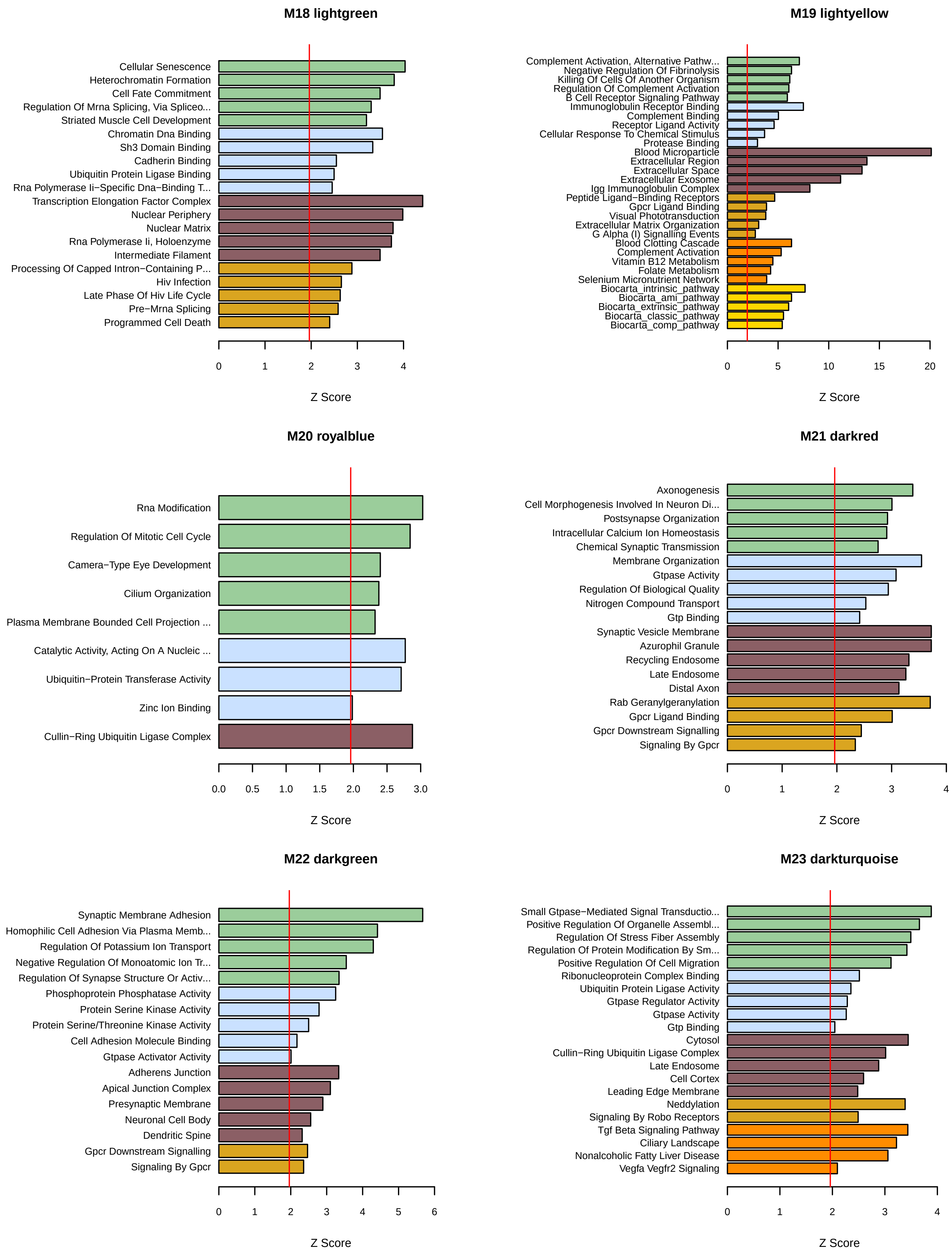
M18 lightgreen
Cellular Senescence
Heterochromatin Formation
Cell Fate Commitment
Regulation Of Mrna Splicing, Via Spliceo...
Striated Muscle Cell Development
Chromatin Dna Binding
Sh3 Domain Binding
Cadherin Binding
Ubiquitin Protein Ligase Binding
Rna Polymerase Ii−Specific Dna−Binding T...
Transcription Elongation Factor Complex
Nuclear Periphery
Nuclear Matrix
Rna Polymerase Ii, Holoenzyme
Intermediate Filament
Processing Of Capped Intron−Containing P...
Hiv Infection
Late Phase Of Hiv Life Cycle
Pre−Mrna Splicing
Programmed Cell Death
0 1 2 3 4
Z Score
M19 lightyellow
Complement Activation, Alternative Pathw...
Negative Regulation Of Fibrinolysis
Killing Of Cells Of Another Organism
Regulation Of Complement Activation
B Cell Receptor Signaling Pathway
Immunoglobulin Receptor Binding
Complement Binding
Receptor Ligand Activity
Cellular Response To Chemical Stimulus
Protease Binding
Blood Microparticle
Extracellular Region
Extracellular Space
Extracellular Exosome
Igg Immunoglobulin Complex
Peptide Ligand−Binding Receptors
Gpcr Ligand Binding
Visual Phototransduction
Extracellular Matrix Organization
G Alpha (I) Signalling Events
Blood Clotting Cascade
Complement Activation
Vitamin B12 Metabolism
Folate Metabolism
Selenium Micronutrient Network
Biocarta_intrinsic_pathway
Biocarta_ami_pathway
Biocarta_extrinsic_pathway
Biocarta_classic_pathway
Biocarta_comp_pathway
0 5 10 15 20
Z Score
M20 royalblue
Rna Modification
Regulation Of Mitotic Cell Cycle
Camera−Type Eye Development
Cilium Organization
Plasma Membrane Bounded Cell Projection ...
Catalytic Activity, Acting On A Nucleic ...
Ubiquitin−Protein Transferase Activity
Zinc Ion Binding
Cullin−Ring Ubiquitin Ligase Complex
0.0 0.5 1.0 1.5 2.0 2.5 3.0
Z Score
M21 darkred
Axonogenesis
Cell Morphogenesis Involved In Neuron Di...
Postsynapse Organization
Intracellular Calcium Ion Homeostasis
Chemical Synaptic Transmission
Membrane Organization
Gtpase Activity
Regulation Of Biological Quality
Nitrogen Compound Transport
Gtp Binding
Synaptic Vesicle Membrane
Azurophil Granule
Recycling Endosome
Late Endosome
Distal Axon
Rab Geranylgeranylation
Gpcr Ligand Binding
Gpcr Downstream Signalling
Signaling By Gpcr
0 1 2 3 4
Z Score
M22 darkgreen
Synaptic Membrane Adhesion
Homophilic Cell Adhesion Via Plasma Memb...
Regulation Of Potassium Ion Transport
Negative Regulation Of Monoatomic Ion Tr...
Regulation Of Synapse Structure Or Activ...
Phosphoprotein Phosphatase Activity
Protein Serine Kinase Activity
Protein Serine/Threonine Kinase Activity
Cell Adhesion Molecule Binding
Gtpase Activator Activity
Adherens Junction
Apical Junction Complex
Presynaptic Membrane
Neuronal Cell Body
Dendritic Spine
Gpcr Downstream Signalling
Signaling By Gpcr
0 1 2 3 4 5 6
Z Score
M23 darkturquoise
Small Gtpase−Mediated Signal Transductio...
Positive Regulation Of Organelle Assembl...
Regulation Of Stress Fiber Assembly
Regulation Of Protein Modification By Sm...
Positive Regulation Of Cell Migration
Ribonucleoprotein Complex Binding
Ubiquitin Protein Ligase Activity
Gtpase Regulator Activity
Gtpase Activity
Gtp Binding
Cytosol
Cullin−Ring Ubiquitin Ligase Complex
Late Endosome
Cell Cortex
Leading Edge Membrane
Neddylation
Signaling By Robo Receptors
Tgf Beta Signaling Pathway
Ciliary Landscape
Nonalcoholic Fatty Liver Disease
Vegfa Vegfr2 Signaling
0 1 2 3 4
Z Score

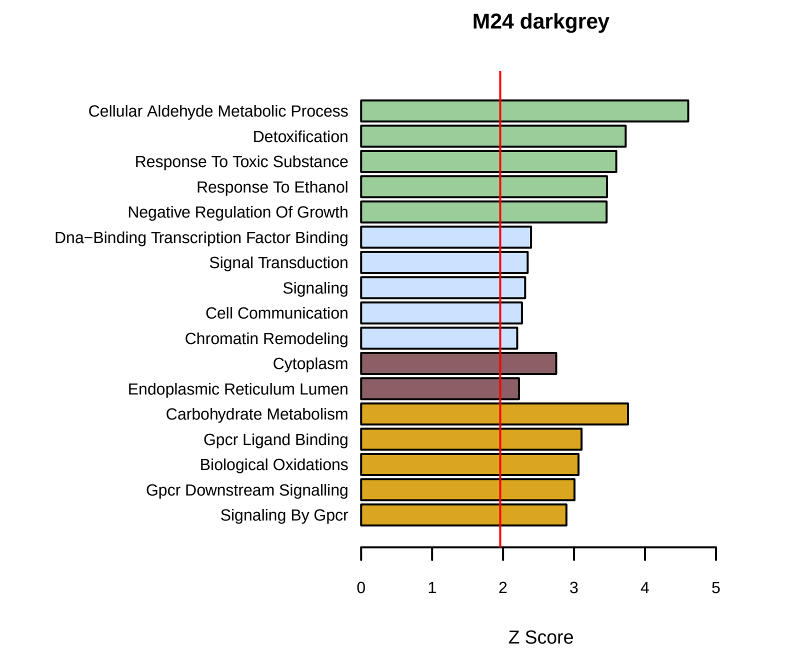
M24 darkgrey
Cellular Aldehyde Metabolic Process
Detoxification
Response To Toxic Substance
Response To Ethanol
Negative Regulation Of Growth
Dna−Binding Transcription Factor Binding
Signal Transduction
Signaling
Cell Communication
Chromatin Remodeling
Cytoplasm
Endoplasmic Reticulum Lumen
Carbohydrate Metabolism
Gpcr Ligand Binding
Biological Oxidations
Gpcr Downstream Signalling
Signaling By Gpcr
0 1 2 3 4 5
Z Score

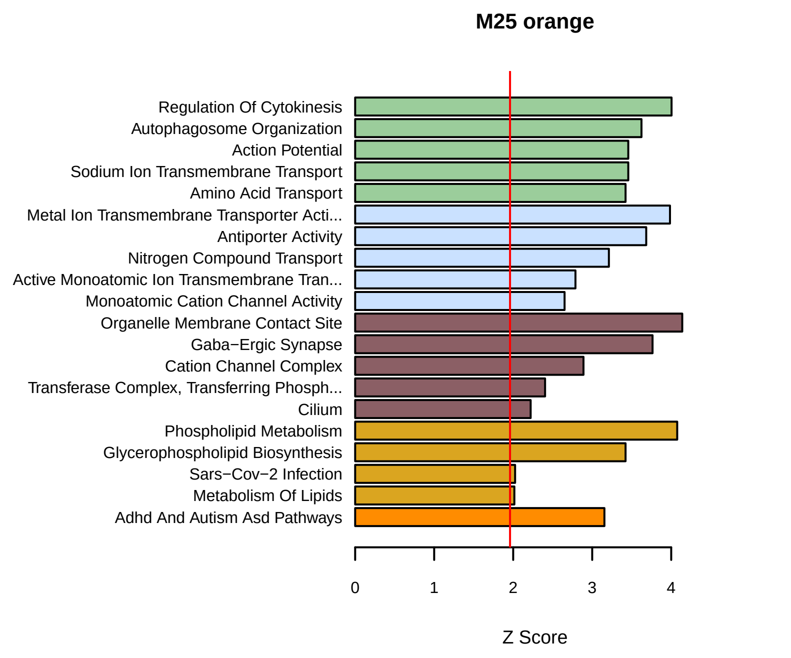
M25 orange
Regulation Of Cytokinesis
Autophagosome Organization
Action Potential
Sodium Ion Transmembrane Transport
Amino Acid Transport
Metal Ion Transmembrane Transporter Acti...
Antiporter Activity
Nitrogen Compound Transport
Active Monoatomic Ion Transmembrane Tran...
Monoatomic Cation Channel Activity
Organelle Membrane Contact Site
Gaba−Ergic Synapse
Cation Channel Complex
Transferase Complex, Transferring Phosph...
Cilium
Phospholipid Metabolism
Glycerophospholipid Biosynthesis
Sars−Cov−2 Infection
Metabolism Of Lipids
Adhd And Autism Asd Pathways
0 1 2 3 4
Z Score

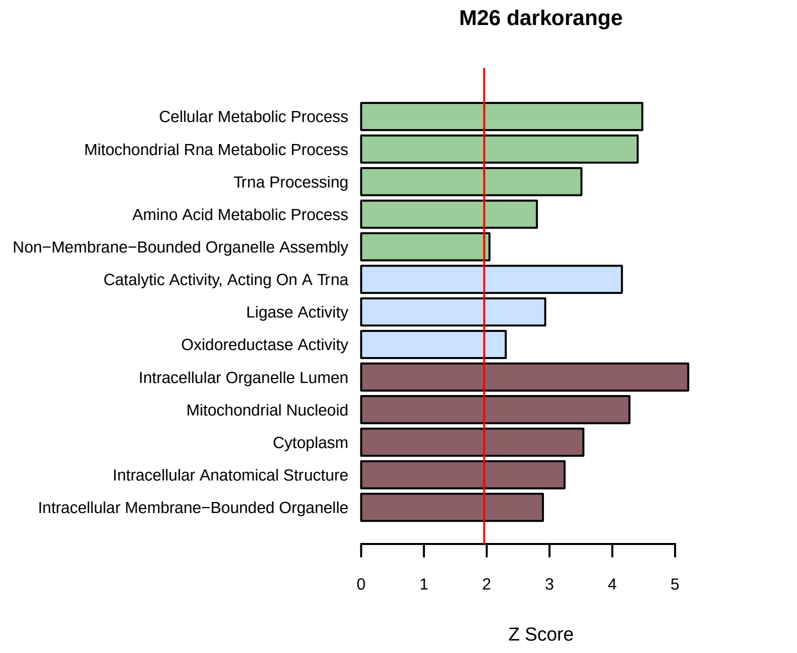
M26 darkorange
Cellular Metabolic Process
Mitochondrial Rna Metabolic Process
Trna Processing
Amino Acid Metabolic Process
Non−Membrane−Bounded Organelle Assembly
Catalytic Activity, Acting On A Trna
Ligase Activity
Oxidoreductase Activity
Intracellular Organelle Lumen
Mitochondrial Nucleoid
Cytoplasm
Intracellular Anatomical Structure
Intracellular Membrane−Bounded Organelle
0 1 2 3 4 5
Z Score

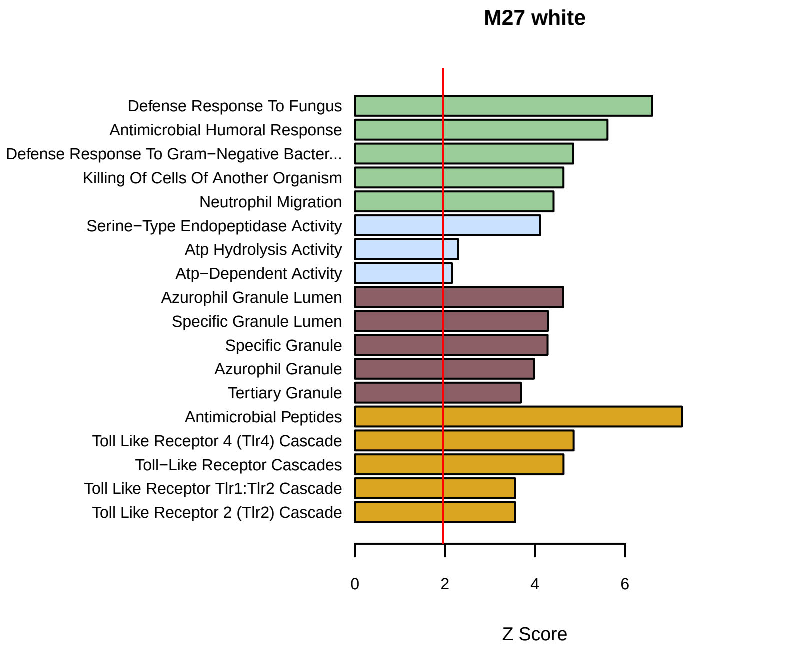
M27 white
Defense Response To Fungus
Antimicrobial Humoral Response
Defense Response To Gram−Negative Bacter...
Killing Of Cells Of Another Organism
Neutrophil Migration
Serine−Type Endopeptidase Activity
Atp Hydrolysis Activity
Atp−Dependent Activity
Azurophil Granule Lumen
Specific Granule Lumen
Specific Granule
Azurophil Granule
Tertiary Granule
Antimicrobial Peptides
Toll Like Receptor 4 (Tlr4) Cascade
Toll−Like Receptor Cascades
Toll Like Receptor Tlr1:Tlr2 Cascade
Toll Like Receptor 2 (Tlr2) Cascade
0 2 4 6
Z Score

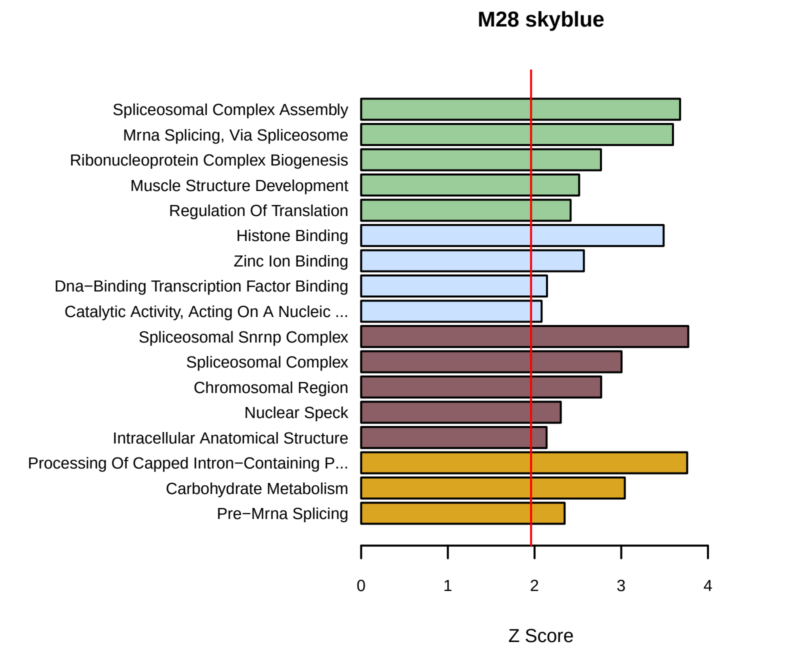
M28 skyblue
Spliceosomal Complex Assembly
Mrna Splicing, Via Spliceosome
Ribonucleoprotein Complex Biogenesis
Muscle Structure Development
Regulation Of Translation
Histone Binding
Zinc Ion Binding
Dna−Binding Transcription Factor Binding
Catalytic Activity, Acting On A Nucleic ...
Spliceosomal Snrnp Complex
Spliceosomal Complex
Chromosomal Region
Nuclear Speck
Intracellular Anatomical Structure
Processing Of Capped Intron−Containing P...
Carbohydrate Metabolism
Pre−Mrna Splicing
0 1 2 3 4
Z Score

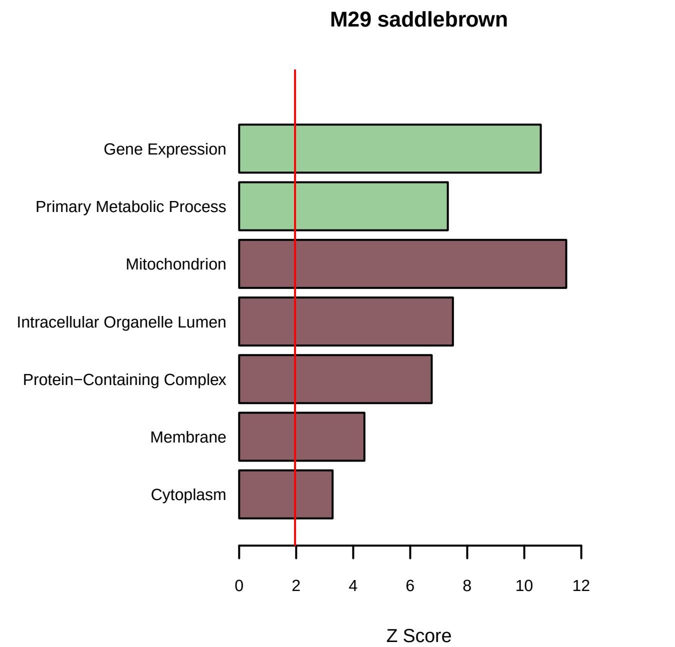
M29 saddlebrown
Gene Expression
Primary Metabolic Process
Mitochondrion
Intracellular Organelle Lumen
Protein−Containing Complex
Membrane
Cytoplasm
0 2 4 6 8 10 12
Z Score

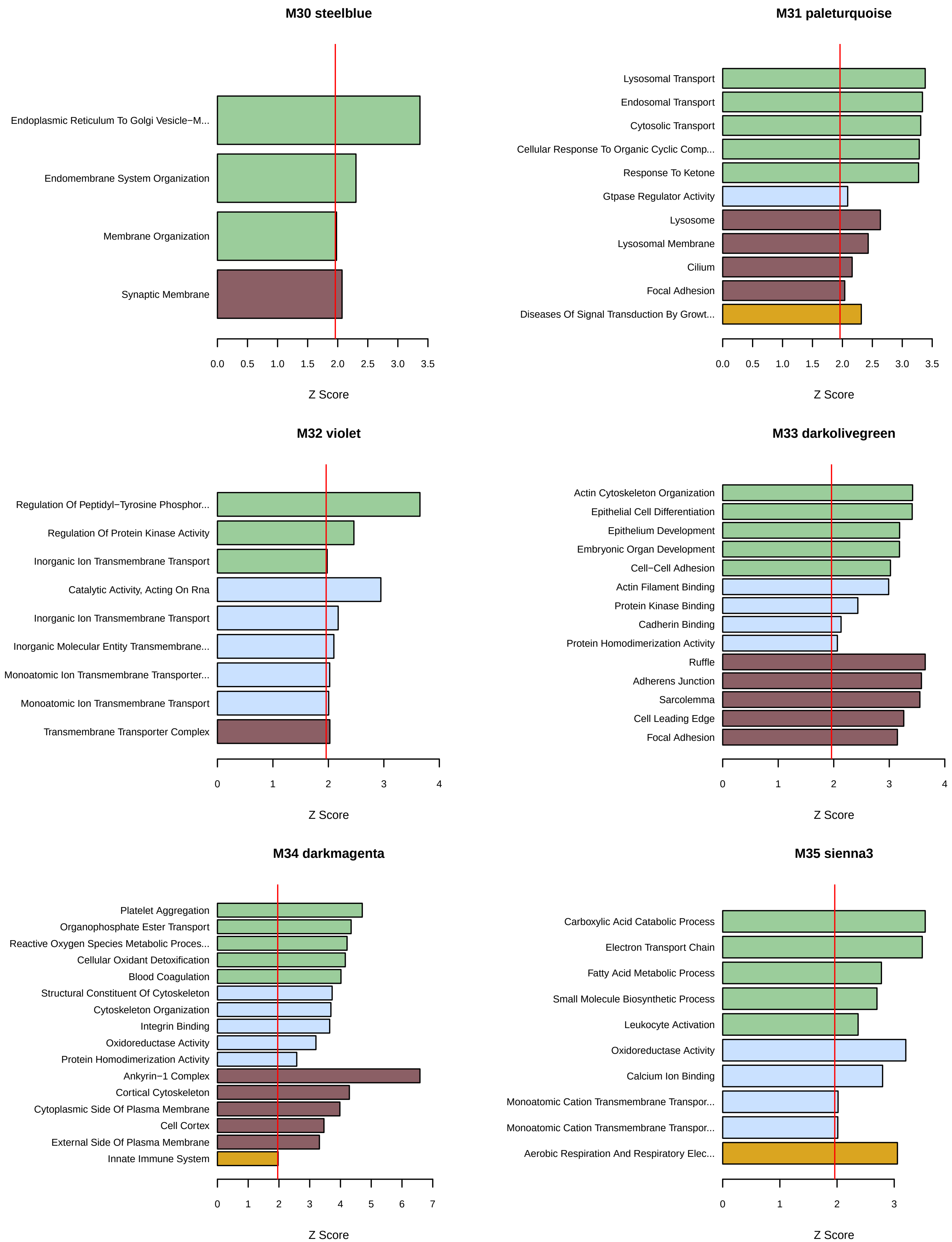
M30 steelblue
Endoplasmic Reticulum To Golgi Vesicle−M...
Endomembrane System Organization
Membrane Organization
Synaptic Membrane
0.0
0.5
1.0
1.5
2.0
2.5
3.0
3.5
Z Score
M31 paleturquoise
Lysosomal Transport
Endosomal Transport
Cytosolic Transport
Cellular Response To Organic Cyclic Comp...
Response To Ketone
Gtpase Regulator Activity
Lysosome
Lysosomal Membrane
Cilium
Focal Adhesion
Diseases Of Signal Transduction By Growt...
0.0
0.5
1.0
1.5
2.0
2.5
3.0
3.5
Z Score
M32 violet
Regulation Of Peptidyl−Tyrosine Phosphor...
Regulation Of Protein Kinase Activity
Inorganic Ion Transmembrane Transport
Catalytic Activity, Acting On Rna
Inorganic Ion Transmembrane Transport
Inorganic Molecular Entity Transmembrane...
Monoatomic Ion Transmembrane Transporter...
Monoatomic Ion Transmembrane Transport
Transmembrane Transporter Complex
0
1
2
3
4
Z Score
M33 darkolivegreen
Actin Cytoskeleton Organization
Epithelial Cell Differentiation
Epithelium Development
Embryonic Organ Development
Cell−Cell Adhesion
Actin Filament Binding
Protein Kinase Binding
Cadherin Binding
Protein Homodimerization Activity
Ruffle
Adherens Junction
Sarcolemma
Cell Leading Edge
Focal Adhesion
0
1
2
3
4
Z Score
M34 darkmagenta
Platelet Aggregation
Organophosphate Ester Transport
Reactive Oxygen Species Metabolic Proces...
Cellular Oxidant Detoxification
Blood Coagulation
Structural Constituent Of Cytoskeleton
Cytoskeleton Organization
Integrin Binding
Oxidoreductase Activity
Protein Homodimerization Activity
Ankyrin−1 Complex
Cortical Cytoskeleton
Cytoplasmic Side Of Plasma Membrane
Cell Cortex
External Side Of Plasma Membrane
Innate Immune System
0
1
2
3
4
5
6
7
Z Score
M35 sienna3
Carboxylic Acid Catabolic Process
Electron Transport Chain
Fatty Acid Metabolic Process
Small Molecule Biosynthetic Process
Leukocyte Activation
Oxidoreductase Activity
Calcium Ion Binding
Monoatomic Cation Transmembrane Transpor...
Monoatomic Cation Transmembrane Transpor...
Aerobic Respiration And Respiratory Elec...
0
1
2
3
Z Score

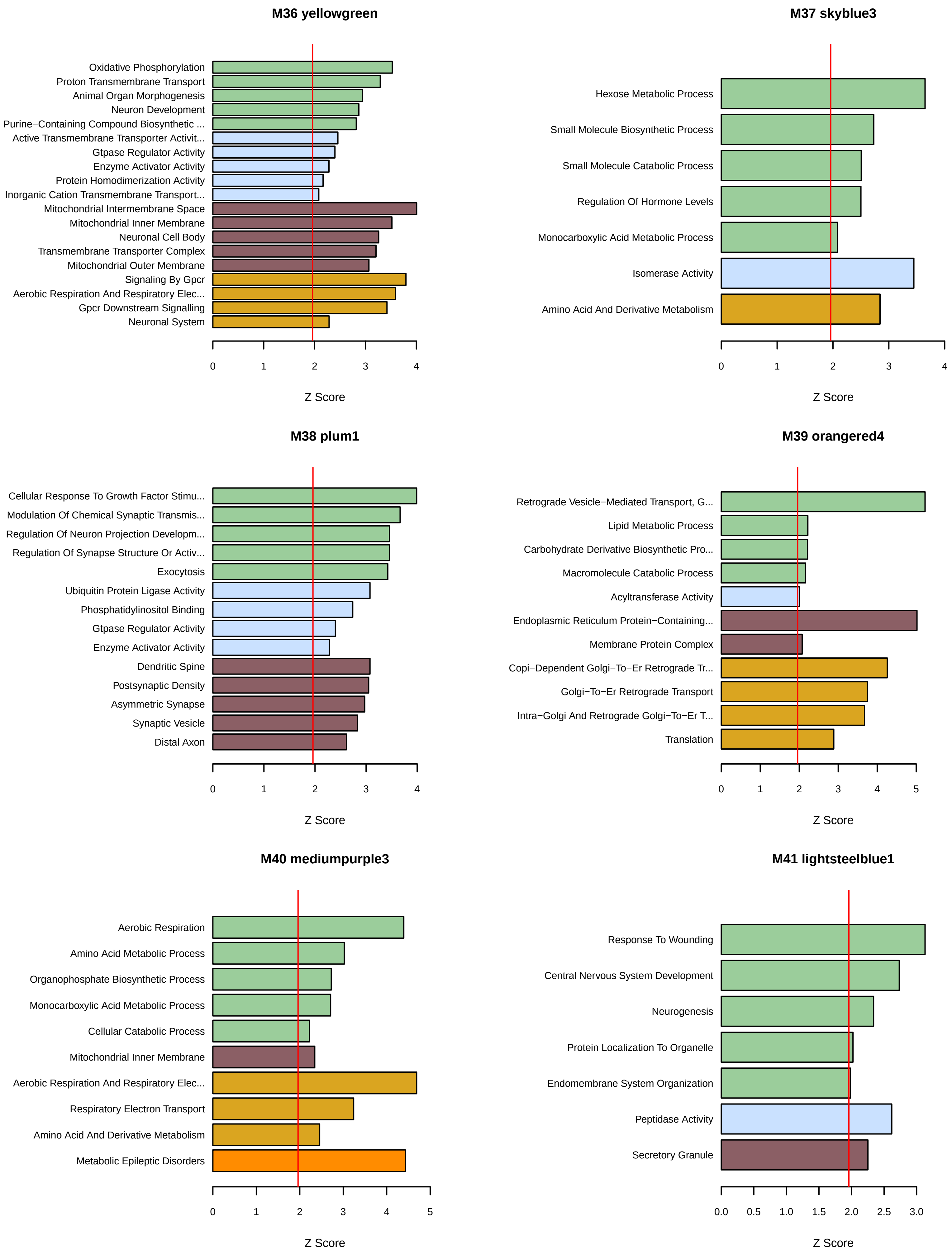
M36 yellowgreen
Oxidative Phosphorylation
Proton Transmembrane Transport
Animal Organ Morphogenesis
Neuron Development
Purine−Containing Compound Biosynthetic ...
Active Transmembrane Transporter Activit...
Gtpase Regulator Activity
Enzyme Activator Activity
Protein Homodimerization Activity
Inorganic Cation Transmembrane Transport...
Mitochondrial Intermembrane Space
Mitochondrial Inner Membrane
Neuronal Cell Body
Transmembrane Transporter Complex
Mitochondrial Outer Membrane
Signaling By Gpcr
Aerobic Respiration And Respiratory Elec...
Gpcr Downstream Signalling
Neuronal System
0 1 2 3 4
Z Score
M37 skyblue3
Hexose Metabolic Process
Small Molecule Biosynthetic Process
Small Molecule Catabolic Process
Regulation Of Hormone Levels
Monocarboxylic Acid Metabolic Process
Isomerase Activity
Amino Acid And Derivative Metabolism
0 1 2 3 4
Z Score
M38 plum1
Cellular Response To Growth Factor Stimu...
Modulation Of Chemical Synaptic Transmis...
Regulation Of Neuron Projection Developm...
Regulation Of Synapse Structure Or Activ...
Exocytosis
Ubiquitin Protein Ligase Activity
Phosphatidylinositol Binding
Gtpase Regulator Activity
Enzyme Activator Activity
Dendritic Spine
Postsynaptic Density
Asymmetric Synapse
Synaptic Vesicle
Distal Axon
0 1 2 3 4
Z Score
M39 orangered4
Retrograde Vesicle−Mediated Transport, G...
Lipid Metabolic Process
Carbohydrate Derivative Biosynthetic Pro...
Macromolecule Catabolic Process
Acyltransferase Activity
Endoplasmic Reticulum Protein−Containing...
Membrane Protein Complex
Copi−Dependent Golgi−To−Er Retrograde Tr...
Golgi−To−Er Retrograde Transport
Intra−Golgi And Retrograde Golgi−To−Er T...
Translation
0 1 2 3 4 5
Z Score
M40 mediumpurple3
Aerobic Respiration
Amino Acid Metabolic Process
Organophosphate Biosynthetic Process
Monocarboxylic Acid Metabolic Process
Cellular Catabolic Process
Mitochondrial Inner Membrane
Aerobic Respiration And Respiratory Elec...
Respiratory Electron Transport
Amino Acid And Derivative Metabolism
Metabolic Epileptic Disorders
0 1 2 3 4 5
Z Score
M41 lightsteelblue1
Response To Wounding
Central Nervous System Development
Neurogenesis
Protein Localization To Organelle
Endomembrane System Organization
Peptidase Activity
Secretory Granule
0.0 0.5 1.0 1.5 2.0 2.5 3.0
Z Score

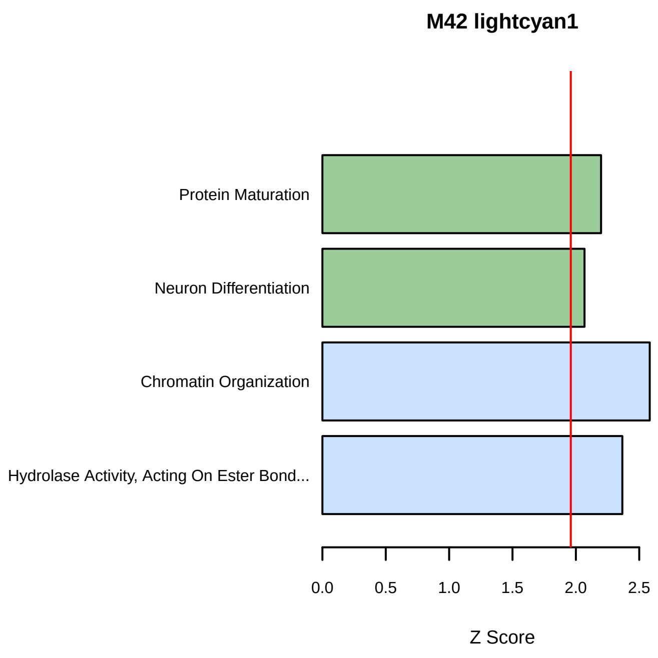
M42 lightcyan1
Protein Maturation
Neuron Differentiation
Chromatin Organization
Hydrolase Activity, Acting On Ester Bond...
0.0
0.5
1.0
1.5
2.0
2.5
Z Score

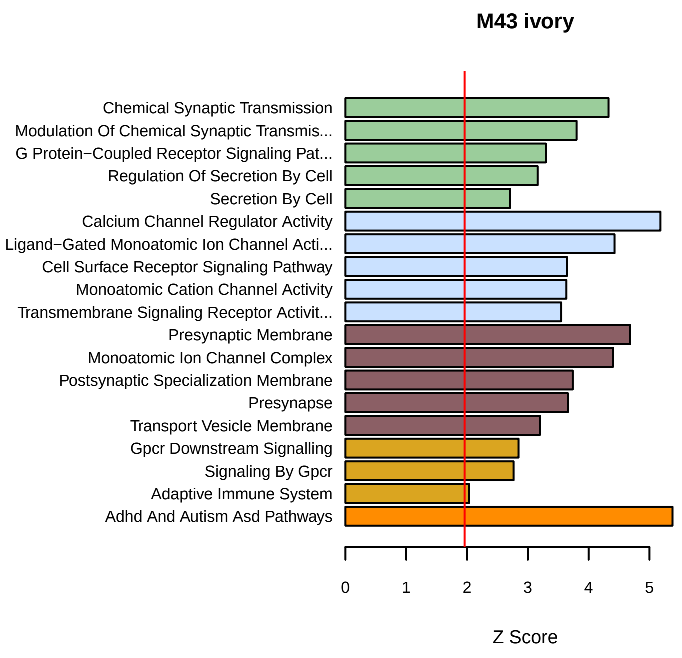
M43 ivory
Chemical Synaptic Transmission
Modulation Of Chemical Synaptic Transmis...
G Protein−Coupled Receptor Signaling Pat...
Regulation Of Secretion By Cell
Secretion By Cell
Calcium Channel Regulator Activity
Ligand−Gated Monoatomic Ion Channel Acti...
Cell Surface Receptor Signaling Pathway
Monoatomic Cation Channel Activity
Transmembrane Signaling Receptor Activit...
Presynaptic Membrane
Monoatomic Ion Channel Complex
Postsynaptic Specialization Membrane
Presynapse
Transport Vesicle Membrane
Gpcr Downstream Signalling
Signaling By Gpcr
Adaptive Immune System
Adhd And Autism Asd Pathways
0
1
2
3
4
5
Z Score
