# Supplementary material for: A Multi-Network Approach Identifies Proteins Related to Dendritic Spines in Alzheimer’s Disease
Source: eNeuro. 2026 Apr 10;13(4):ENEURO.0468-25.2026. doi: 10.1523/ENEURO.0468-25.2026 (PMC13095402; doi:10.1523/ENEURO.0468-25.2026)

Extended Data Figure 3-1. Partition Similarity Heatmaps. Partition Comparisons were performed to assess the similarity between WGCNA and SE2 protein clusters.

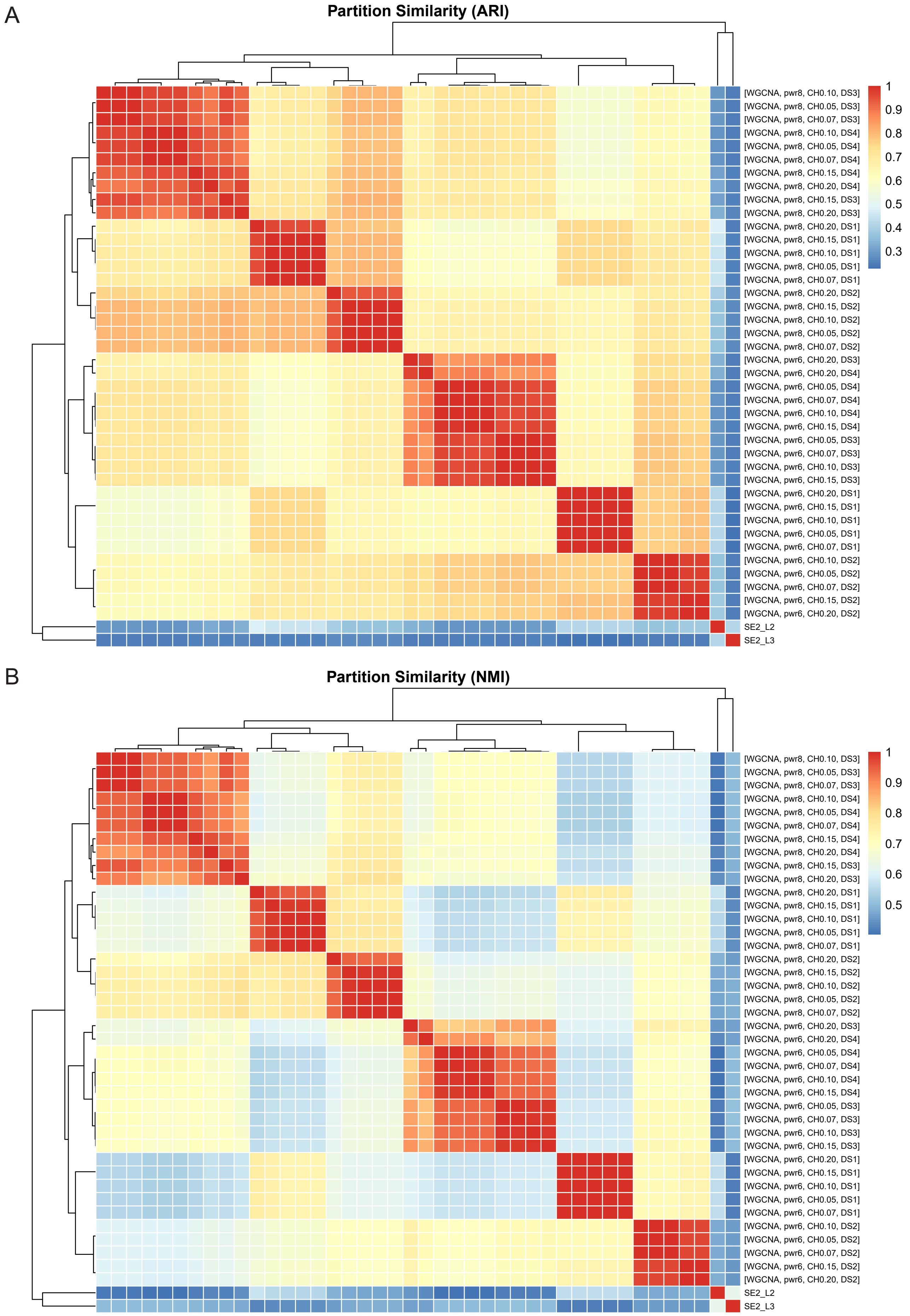

Supplement: Figure 3-1 — Partition Comparison Heatmaps. Partition Comparisons were performed to assess the similarity between WGCNA and SE2 protein clusters. Download Figure 3-1, ZIP file. [file eneuro-13-ENEURO.0468-25.2026-s009.zip › Extended Data Figure 3-1.pdf]
