# Supplementary material for: A Multi-Network Approach Identifies Proteins Related to Dendritic Spines in Alzheimer’s Disease
Source: eNeuro. 2026 Apr 10;13(4):ENEURO.0468-25.2026. doi: 10.1523/ENEURO.0468-25.2026 (PMC13095402; doi:10.1523/ENEURO.0468-25.2026)
Supplement: Figure 3-4 — SE2 GO terms. Gene ontology (GO) analysis was performed to gain insight into the biological meaning of each protein network module. Enrichment for a given ontology is shown by z score. Download Figure 3-4, ZIP file. [file eneuro-13-ENEURO.0468-25.2026-s012.zip › Extended Data Figure 3-4.pdf]

Extended Data Figure 3-4. GO Analysis on SE2 Network Modules. Gene ontology (GO) analysis was performed to gain insight into the biological meaning of each protein network module. Enrichment for a given ontology is shown by z score.

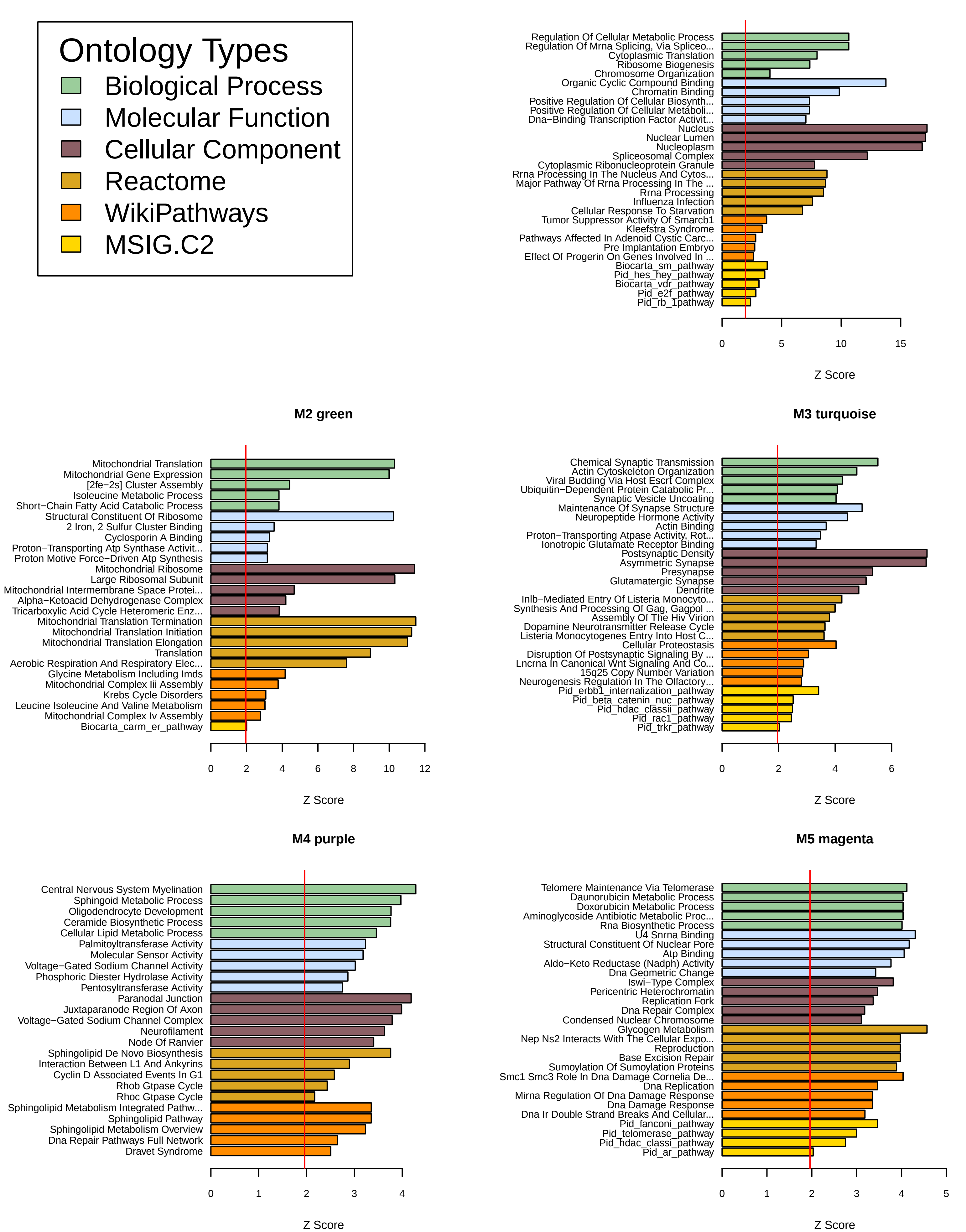

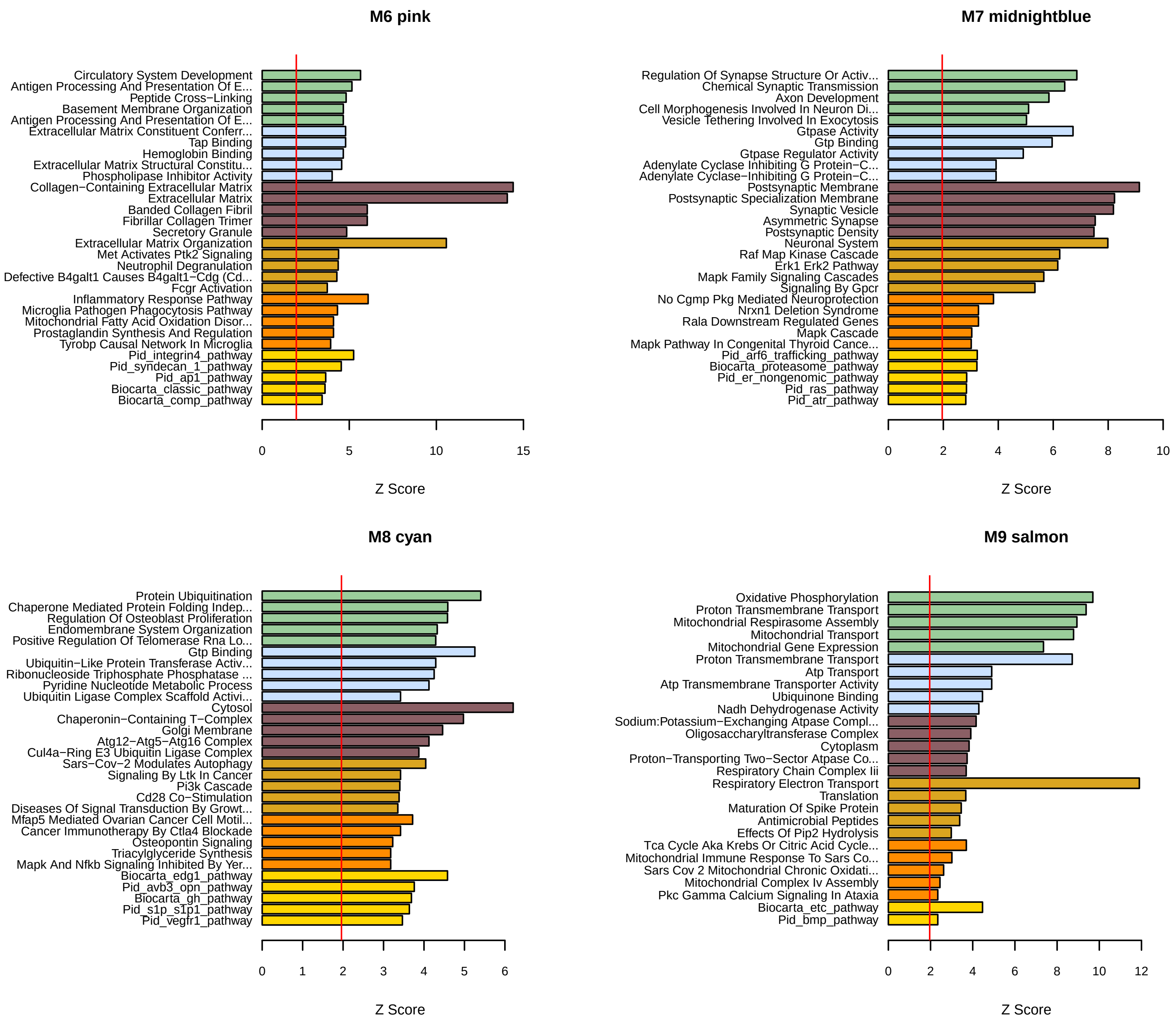
M6 pink
Circulatory System Development
Antigen Processing And Presentation Of E...
Peptide Cross−Linking
Basement Membrane Organization
Antigen Processing And Presentation Of E...
Extracellular Matrix Constituent Conferr...
Tap Binding
Hemoglobin Binding
Extracellular Matrix Structural Constitu...
Phospholipase Inhibitor Activity
Collagen−Containing Extracellular Matrix
Extracellular Matrix
Banded Collagen Fibril
Fibrillar Collagen Trimer
Secretory Granule
Extracellular Matrix Organization
Met Activates Ptk2 Signaling
Neutrophil Degranulation
Defective B4galt1 Causes B4galt1−Cdg (Cd...
Fcgr Activation
Inflammatory Response Pathway
Microglia Pathogen Phagocytosis Pathway
Mitochondrial Fatty Acid Oxidation Disor...
Prostaglandin Synthesis And Regulation
Tyrobp Causal Network In Microglia
Pid_integrin4_pathway
Pid_syndecan_1_pathway
Pid_ap1_pathway
Biocarta_classic_pathway
Biocarta_comp_pathway
0
5
10
15
Z Score
M7 midnightblue
Regulation Of Synapse Structure Or Activ...
Chemical Synaptic Transmission
Axon Development
Cell Morphogenesis Involved In Neuron Di...
Vesicle Tethering Involved In Exocytosis
Gtpase Activity
Gtp Binding
Gtpase Regulator Activity
Adenylate Cyclase Inhibiting G Protein−C...
Adenylate Cyclase−Inhibiting G Protein−C...
Postsynaptic Membrane
Postsynaptic Specialization Membrane
Synaptic Vesicle
Asymmetric Synapse
Postsynaptic Density
Neuronal System
Raf Map Kinase Cascade
Erk1 Erk2 Pathway
Mapk Family Signaling Cascades
Signaling By Gpcr
No Cgmp Pkg Mediated Neuroprotection
Nrxn1 Deletion Syndrome
Rala Downstream Regulated Genes
Mapk Cascade
Mapk Pathway In Congenital Thyroid Cance...
Pid_arf6_trafficking_pathway
Biocarta_proteasome_pathway
Pid_er_nongenomic_pathway
Pid_ras_pathway
Pid_atr_pathway
0
2
4
6
8
10
Z Score
M8 cyan
Protein Ubiquitination
Chaperone Mediated Protein Folding Indep...
Regulation Of Osteoblast Proliferation
Endomembrane System Organization
Positive Regulation Of Telomerase Rna Lo...
Gtp Binding
Ubiquitin−Like Protein Transferase Activ...
Ribonucleoside Triphosphate Phosphatase ...
Pyridine Nucleotide Metabolic Process
Ubiquitin Ligase Complex Scaffold Activi...
Cytosol
Chaperonin−Containing T−Complex
Golgi Membrane
Atg12−Atg5−Atg16 Complex
Cul4a−Ring E3 Ubiquitin Ligase Complex
Sars−Cov−2 Modulates Autophagy
Signaling By Ltk In Cancer
Pi3k Cascade
Cd28 Co−Stimulation
Diseases Of Signal Transduction By Growt...
Mfap5 Mediated Ovarian Cancer Cell Motil...
Cancer Immunotherapy By Ctla4 Blockade
Osteopontin Signaling
Triacylglyceride Synthesis
Mapk And Nfkb Signaling Inhibited By Yer...
Biocarta_edg1_pathway
Pid_avb3_opn_pathway
Biocarta_gh_pathway
Pid_s1p_s1p1_pathway
Pid_vegfr1_pathway
0
1
2
3
4
5
6
Z Score
M9 salmon
Oxidative Phosphorylation
Proton Transmembrane Transport
Mitochondrial Respirasome Assembly
Mitochondrial Transport
Mitochondrial Gene Expression
Proton Transmembrane Transport
Atp Transport
Atp Transmembrane Transporter Activity
Ubiquinone Binding
Nadh Dehydrogenase Activity
Sodium:Potassium−Exchanging Atpase Compl...
Oligosaccharyltransferase Complex
Cytoplasm
Proton−Transporting Two−Sector Atpase Co...
Respiratory Chain Complex Iii
Respiratory Electron Transport
Translation
Maturation Of Spike Protein
Antimicrobial Peptides
Effects Of Pip2 Hydrolysis
Tca Cycle Aka Krebs Or Citric Acid Cycle...
Mitochondrial Immune Response To Sars Co...
Sars Cov 2 Mitochondrial Chronic Oxidati...
Mitochondrial Complex Iv Assembly
Pkc Gamma Calcium Signaling In Ataxia
Biocarta_etc_pathway
Pid_bmp_pathway
0
2
4
6
8
10
12
Z Score
